# Did climate determine Late Pleistocene settlement dynamics in the Ach Valley, SW Germany?

**DOI:** 10.1371/journal.pone.0215172

**Published:** 2019-05-02

**Authors:** Sara E. Rhodes, Britt M. Starkovich, Nicholas J. Conard

**Affiliations:** 1 Institut für Naturwissenschaftliche Archäologie, Universität Tübingen, Tübingen, Germany; 2 Senckenberg Centre for Human Evolution and Palaeoenvironment, Universität Tübingen, Tübingen, Germany; 3 Abteilung Ältere Urgeschichte und Quartärökologie, Universität Tübingen, Tübingen, Germany; Max Planck Institute for the Science of Human History, GERMANY

## Abstract

The loss of Neanderthal groups across Western and Central Europe during Oxygen Isotope Stage (OIS) 3 has held the attention of archaeologists for decades. The role that climatic change, genetic interbreeding, and interspecies competition played in the extinction of Neanderthal groups is still debated. Hohle Fels is one of several important Middle and Upper Paleolithic sites from the Ach Valley in southwestern Germany which documents the presence of Neanderthals and modern humans in the region. Chronological and stratigraphic records indicate that these two groups occupied the site with little to no overlap or interaction. This provides the opportunity to examine the behavioural variability of Swabian Neanderthal populations without the complication of cross-cultural influence. In this study we contribute a terrestrial paleoenvironmental record derived from the small mammal material from Hohle Fels Cave to the ever-growing archaeological record of this period. By reconstructing the climate and landscape of the Ach Valley during this time we can identify the effect that the OIS 3 environment had on the presence of Neanderthals in the region. Based on indicator taxa and the habitat weighing method, the small mammal record, which includes rodents, insectivores, and bats, from Hohle Fels shows that the earliest Neanderthal occupation took place on a landscape characterized by substantial woodland and forest, rivers and ponds, as well as moist meadows and grasslands. A gradual increase in cold tundra and arctic environments is clear towards the end of the Middle Paleolithic, extending to the end of the early Aurignacian which may correlate with the onset of the Heinrich 4 event (~42,000 kya). Our taphonomic analysis indicates the material was accumulated primarily by opportunistic predators such as the great grey owl, snowy owl, and European eagle owl, and therefore reflects the diversity of landscapes present around the site in the past. Importantly, at the time Neanderthals abandoned the Ach Valley we find no indication for dramatic climatic deterioration. Rather, we find evidence of a gradual cooling of the Swabian landscape which may have pushed Neanderthal groups out of the Ach Valley prior to the arrival of modern human Aurignacian groups.

## Introduction

The climatically unstable oxygen isotope stage (OIS) 3 and the extinction of Neanderthals across most of Western and Central Europe during this period has been an important area of archaeological research throughout the 20^th^ century. The Pleistocene record of central and southern Germany includes both cave and open-air sites from OIS 3, which dates roughly from 60,000 to 20,000 years before present (BP). The nature of the sites (i.e. repeat occupations [1] vs. possible temporary mass hunting camps [2]), as well as the duration of occupation varies through time and space. This variability might reflect demographic changes driven by repeated northern advances and southern retreats, shifting subsistence and/or mobility strategies, or the effects of localized extinctions of Neanderthal groups following shifts in glacial ice coverage and arctic tundra during stadial and interstadial events [3,4]. Although issues of site preservation confound the picture, particularly with regard to open-air sites, the record from the beginning of OIS 3 also includes examples of site clusters within small geographic regions. An iconic example of such a cluster of Middle Paleolithic (MP) sites is found in the Swabian Jura.

**Fig 1:**
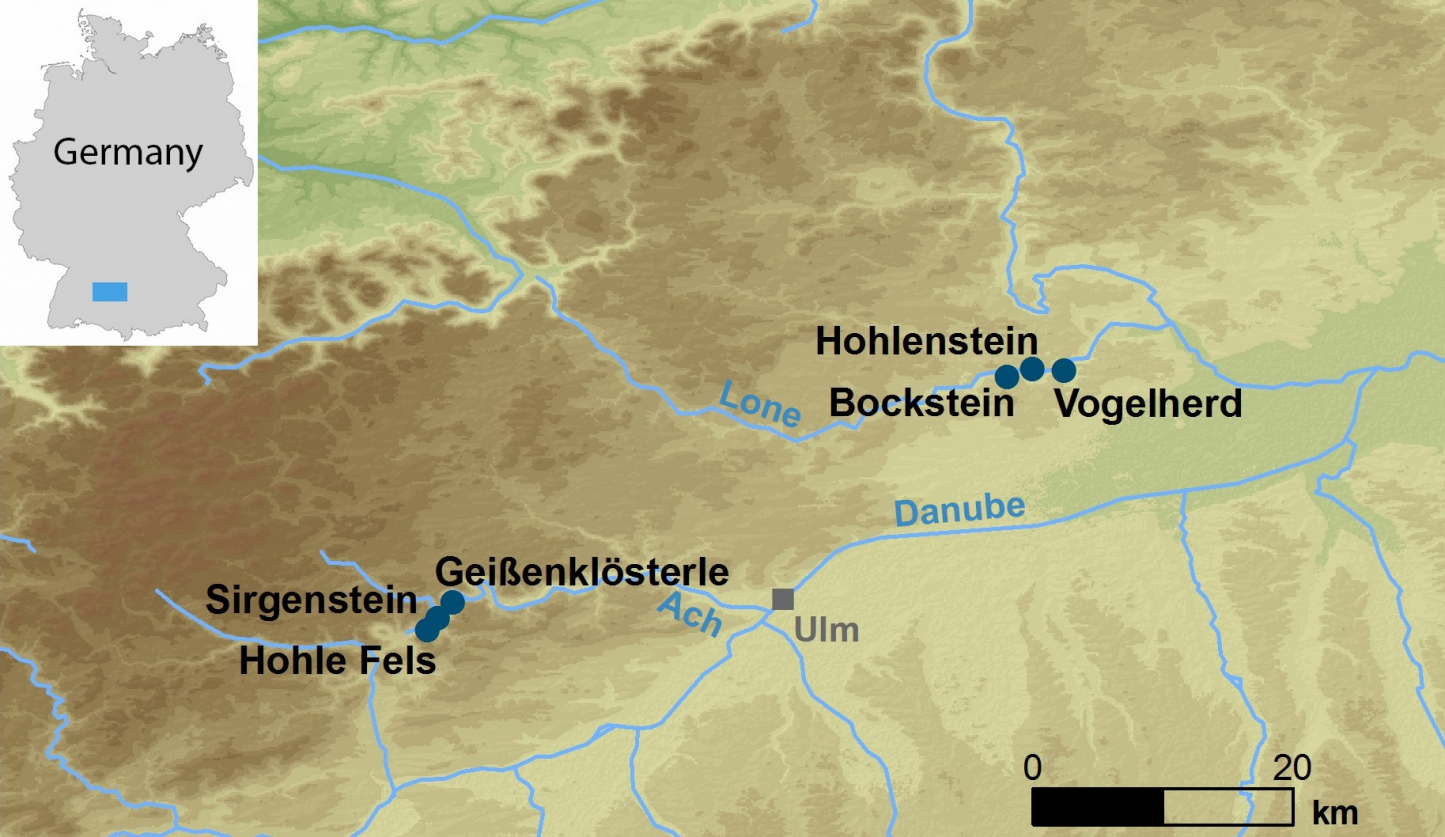
Map of Swabian Alb showing all Paleolithic cave sites mentioned in the text.

The Ach and Lone valleys (Fig 1), located along two tributaries of the Danube in the Swabian Jura, house a number of caves and rock shelters preserving an archaeological record spanning the late MP through to the Neolithic [5]. Many of these sites, such as Geißenklösterle, Vogelherd, and Hohlestein-Stadel are well-known for the spectacular bone and ivory figurines and flutes found in the Aurignacian deposits [6–8]. Others, while lacking such ample evidence for artistic expression, have none-the-less produced important records of both MP and Upper Paleolithic (UP) occupation, including detailed faunal, geoarchaeological, technological and chronological records (i.e. Sirgenstein [5], Große Grotte [9], Kogelstein [10,11], Brillenhöhle [12], and the Bockstein complex [13]). One of the most well-known sites in the Swabian Jura is Hohle Fels, located in the Ach Valley just outside of the town of Schelklingen. Excavations under Nicholas Conard’s direction have continued annually for more than 20 years and have produced a rich material record spanning the late MP through to the Mesolithic, including symbolic artifacts such as ivory animal and human figurines [14], musical instruments [6], and one of the oldest phallic representations in the world [15].

Despite clear evidence of occupation by both MP (i.e. Neanderthal) and UP (i.e. anatomically modern human) groups, the chronological and stratigraphic records of many sites from the Ach and Lone valleys strongly suggest that a temporal hiatus separates these occupations [16,17]. The clearest indications of this hiatus is seen in the geogenic deposits that typically separate the final MP and the earliest Aurignacian [5,17]. Where these layers are clearly stratified and easily delineated, as at Geißenklösterle and Hohle Fels, they can be used to test hypotheses related to the depopulation of the region by Neanderthal groups prior to modern human arrival [18].

Numerous hypotheses have been proposed, and in some cases clearly refuted [19], to explain the loss of Neanderthal groups across Europe around the same time the continent was first occupied by modern humans. Some researchers now support a multi-faceted model for Neanderthal extinction, which includes competition [20,21] and interbreeding with modern human groups [22] in combination with dramatic climatic change, small population size, and genetic bottlenecks [19], as key forces driving Neanderthal groups into refugia and triggering population collapse [19,21,23,24]. Understanding why Neanderthals may have chosen to abandon regions they occupied repeatedly over extended periods may provide important insight into the species’ adaptive strategies, particularly in relation to group mobility. Areas like the Swabian Jura and elsewhere along the upper and middle Danube [25], where the archaeological record suggests interactions between Neanderthals and modern human groups did not occur, provide the opportunity to explore Neanderthal mobility and behavioural variability in the absence of cross-cultural influence [alternatively see 26]. Establishing the impact of external forces, such as climatic instability, during the final period of Neanderthal occupation of a region is a key step in this line of inquiry.

To this end, we present here a detailed climatic record derived from our analysis of the small mammal material from Hohle Fels designed to identify periods of dramatic climatic shifts correlating with the final occupation of the Ach Valley by Neanderthal groups. In applying a modified indicator species method [18] and the habitat weighting method [27,28] to material recovered from deposits dating from ≥ 44,300 calBP to 39,000 calBP [29], we have identified changes in the vegetation of the Ach Valley landscape and broad shifts in the temperature and humidity of the region. Contextualizing this record within the history of paleoenvironmental research at Hohle Fels and Geißenklösterle allows us to test environmentally driven models for the depopulation of the Ach Valley.

## Site description

The Swabian Jura is a karstic plateau formed primarily of Jurassic limestone which ranges from 500 to 1500 m a.s.l. and extends across part of southwestern Germany, with the Neckar Valley to the north and the Danube Valley to the south [30]. The Ach and Lone valleys, formed by tributaries of the Danube River, include a number of important archaeological cave sites with MP and UP deposits. Hohle Fels (48°22'45''N E9°45'14''E) sits at 534 m a.s.l. and comprises a 29 meter long corridor and a 23 m long main hall, both of which were filled with sediments deposited primarily through a now closed chimney at the back of the cave [30,31]. The cave entrance opens to the northwest and overlooks the Ach Valley, which is now filled with fluvial sediments and lies 5–10 m higher than in the Late Pleistocene [30]. Archaeological excavation of the cave has been ongoing since 1977 by the University of Tübingen under the direction of Joachim Hahn and subsequently Nicholas Conard. However, the earliest investigations occurred between 1870 and 1871 and were conducted by Oscar Fraas and Theodor J. Hartmann [5]. Gustav Riek also directed excavations from 1958 to 1960, however his results were never published.

**Fig 2:**
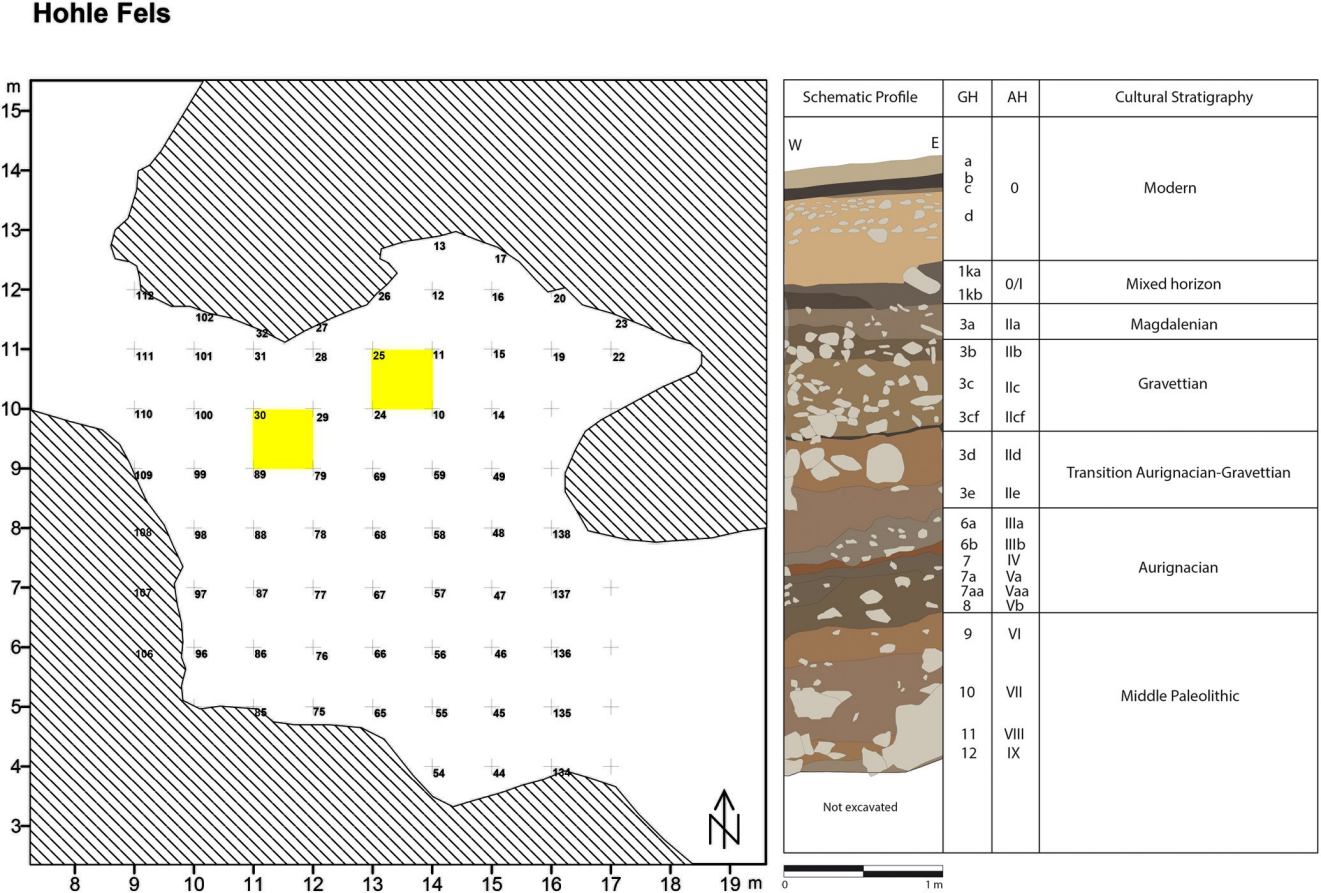
Excavation grid of Hohle Fels (left) with quadrants 30 and 25 highlighted; Stratigraphic profile (right) with correlated geological horizons (GH) and archaeological horizons (AH) and cultural periods.

The combined excavations by Hahn and Conard have produced a 5-meter-thick stratigraphic sequence spanning the Holocene through to the MP. The stratigraphic record is divided into geological horizons (GHs) indicated by Arabic numerals and archaeological horizons (AHs) indicated by Roman numerals with letter subdivisions which often, but not always, correspond to each other (Fig 2). Aurignacian lithic and symbolic materials are found from GH 6a through GH 8, while MP lithic artifacts occur in much lower densities from GH 9 through GH 12. A possible cultural hiatus has been identified in the lower portion of GH 8, akin to the nearly-culturally sterile horizons separating MP and UP deposits at Geißenklösterle [31], Vogelherd, [31] and Sirgenstein [5] caves.

## Materials and methods

### Sample selection

The small mammal material from Hohle Fels analyzed for this study derived from two 1 m^2^ excavation units (Qu. 30 and 25) and includes GH 7 through 12. Excavation permits were granted by the State Heritage office of Baden-Württemberg (Baden-Württemberg Landsamt für Denkmalpflege) and the specimen collections are accessibly housed at the Universität Tübingen Institut für Naturwissenschaftliche Archaëologie in Tübingen, Germany. No permits were required for the described study.

Due to similarities in the geological substrate of GH 7a and 7aa [31,32], these stratigraphic units have been grouped as 7a/7aa for the current study. Combining these layers also results in a taxonomic sample size large enough to allow statistical analysis, which greatly enhances the informative potential of the material. The exceptional quality and density of anthropogenic finds recovered from Qu. 30 include numerous lithic and bone tools, ivory and bone flute fragments [33] and the Venus figurine [34]. Excavation of Qu. 30 has not yet reached the earliest MP, therefore material from GH 11 and 12 originating in Qu. 25 was also included in this study. Qu. 25 was chosen for its extremely high quantity of small mammal remains and its close proximity to Qu. 30.

### Taxonomic methods

At Hohle Fels, sediments are excavated from 25 cm^2^ sub-quadrants and water-screened on site using water pumped from the nearby Ach River. The remaining material is then sorted in the field house in Blaubeuren by material type and size. All bones, including fragments, are removed and sorted by either specimen size or broad taxonomic category, which include bird, fish, and microfauna. The small mammal material is then sorted from the amphibian, reptile, and small bird and fish remains in the zooarchaeology lab at the University of Tübingen. A Euromax binocular microscope is used to examine all specimens at 10x – 50x magnification. Our taxonomic identification focused on isolated dental elements (maxillae, mandibles, incisors and molars) and select post-cranial elements via direct comparison with osteological collections at the University of Tübingen and published morphological and metric data including Adadjanian and von Koenigswald [35], van Kolfschoten [36] and Nadachowski [37] for Arvicolidae, Corbet [38] for Gliridae, Ziegler [39] for Talpidae, Popova [40,41] and Cubuk [42] for Sciuridae, Reumer [43] and Heinrich [44] for Soricidae, and Storch [12] and Sevilla [45] for Chiroptera. We identified arvicolid taxa by a combination of lower m1 occlusal morphology and occlusal measurements which follow van Kolfschoten [36]. *Dicrostonyx gulielmi* is the only exception, as these specimens are identified to genus based on the combination of tooth morphology, a lack of enamel along the triangle salient edges, and the lack of cementum in re-entrant angles. Species designations are based on the abundance of Agadjanian & von Koenigswald’s [35] morphotype 1 and 2 in the assemblage.

We calculated diversity indices including richness (NTAXA), and heterogeneity (reciprocal of Simpson’s Index, 1/D [46]). We measured NTAXA as the total number of taxonomic designations below family level (including genera identifications only when species-level attributions were not possible). The reciprocal of Simpson’s Index (1/D) was calculated as

$$1/D=\Sigma p_{i}{}^{2}$$

Where *p* is the proportional abundance of taxon *i*. This measure is expressed here as 1/D so that greater evenness is suggested by a larger value, with the minimum possible value being one and the maximum being the NTAXA in the assemblage. To assess the degree to which the small mammal material from Hohle Fels suffers from a sampling bias limiting the representation of rare species [47], we produced rarefaction curves using NTAXA data for each geological horizon in PAST 3.14.

### Taphonomic methods

Conducting detailed taphonomic analyses of all faunal remains recovered from archaeological contexts is an important first step before interpreting evidence of past environments or site specific human behaviours. To this end, we conducted a full taphonomic analysis of both the crania and post-crania of the small mammal material to identify the mode of accumulation and to qualify any related taxonomic bias. We documented the presence, absence, or degree of pronouncement (as a categorical ranking) of bone and tooth modifications including fragmentation, surface cracking, edge rounding, root etching, abrasion, weathering, thermal discolouration, oxide staining, and digestive corrosion. Identification of these modifications was based on Andrews [48], Fernandez-Jalvo & Andrews [49], Madgwick [50], Marín-Arroyo et al. [51], and Stiner et al. [52].

Description of the fragmentation of cranial and post-cranial elements follows Andrews’ [48] breakage classes. We calculated relative abundances for each skeletal element by GH using the formula *R* = *N_i_*/(*MNI x E_i_*), with E_i_ being the number of element *i* expected in a single prey skeleton and MNI being the minimum number of individuals based on the most abundant skeletal element within the GH assemblage. This weighted abundance is used to distinguish between predator produced prey accumulations in microfaunal studies [49,53–55]. We applied a correlation coefficient, Kendall’s Tau, to compare our relative element abundances with Andrews’ [48] actualistic data. The high possibility that the small mammal skeletal remains from Hohle Fels were subjected to substantial post-depositional breakage through non-predator agents such as sediment compression and trampling limits the interpretive potential of the skeletal element representation data. This was noted by Andrews [48] as a complicating factor when studying material from taphonomically complex contexts. To correct for this, we also documented digestive corrosion on incisors, molars and select post-cranial elements throughout the Hohle Fels material and compared this with Andrews’ [48] actualistic results.

Digestive corrosion of both mandibular and maxillary incisors presents on either the tip or the anterior surface of the tooth, and we documented the occurrence and intensity of this modification separately for both portions. The progressive nature of digestive destruction also follows tooth shape and therefore presents differently for arvicolid, murid and soricid molar dentition [56]. Only two Soricidae specimens exhibited digestive corrosion, and in each case the modification was of the lowest intensity category (moderate) for these taxa. Therefore, only arvicolid digestive corrosion is reported herein. The categorical ranking of modification intensity is the same as that used for incisors, with progressive enamel damage scaled from light to extreme.

Andrews [48] describes two types of bone modification due to exposure to digestive acids: intrusive digestion on the articular ends and epiphyseal fusion lines and rounding of broken edges and nearby bone surfaces. This second type of bone modification is equifinal with a number of post-depositional processes (such as weathering, soil acid corrosion, sediment abrasion and tumbling damage), and is therefore less informative of predator action and not used in this study. As digestive corrosion is heavier on less mineralized juvenile epiphyses and metaphyses intrusive digestion has been documented on fully fused adult bone, only.

### Paleoenvironmental reconstruction methods

We used both the modified indicator taxa method [27] and the Habitat Weighting method [28] to interpret the paleoenvironmental signal of the small mammal material from Hohle Fels. When applying the modified indicator taxa method, we assigned each taxon a vegetative and climatic niche based on the habitat preferences of published modern German analogues [48,57,58] and those derived from paleontological studies [1,37,59,60]. A list of the species present in the Hohle Fels assemblage and their identified niches is included in Table 1.We then calculated the proportion of individuals allocated to each vegetative/climatic niche out of the total sample for each geological horizon and compared these percentages within and between horizons to identify shifts in the environmental pattern between key archaeological periods and through time generally [18]. Comey et al. [61] have shown that this method, which is also referred to as the taxonomic abundance method [62], is highly informative when the environmental variables considered are those that direct small mammal community structure, particularly local vegetative structure. Additionally, these authors [61] found that predator misidentification lead to erroneous environmental signals in their study of modern small mammal assemblages. This assumedly holds true in archaeological studies, as well, and supports the inclusion of a complete taphonomic analysis in this study.

**Table 1:** List of species and primary habitat preference used for the indicator species analysis of the Hohle Fels Cave small mammal assemblage.

Species	Habitat Preference
**Insectivora**	
*Crocidura leucodon/russula*	Open forest, warm and dry
*Sorex minutus*	Open forest, cool and moist
*Sorex araneus*	Open forest, cool and moist
*Neomys fodiens*	Open forest, cool and moist
**Rodentia**	
Sciuridae	
*Spermophilus superciliosus*	Steppe grassland
Arvicolidae	
*Lemmus lemmus*	Cold tundra
*Dicrostonyx gulielmi*	Cold tundra
*Microtus gregalis*	Cold tundra/wooded steppe
*Chionomys nivalis*	Rocky
*Microtus oeconomus*	Boreal forest
*Arvicola terrestris/antiquus*	Open forest and lake margins
*Microtus subterraneus*	Indeterminate
*Microtus arvalis/agrestis*	Indeterminate

Additionally, we employed the habitat weighing method [28] which distributes each small mammal species into habitat types based on their modern distributions. Six habitat types were represented in the Hohle Fels small mammal assemblage including open dry meadows (OD), open, humid evergreen meadows with dense pastures (OH), open woodland and forest edges (OW), mature forest woodlands (Wo), rocky areas with stony substratum (Rocky) and areas along streams, lakes and pond edges (Water). We added two additional habitat types as the assemblage includes species found exclusively in or partly within cold, dry treeless landscapes which may have permanently frozen subsoils (Tundra) and temperate grasslands (Steppe). This method differs from the indicator taxa method as it takes into account the fact that some species may inhabit multiple habitat types when available on the landscape. The weighting (1.0) for species with such broad tolerances is distributed across multiple habitat types according to how often they are found within each habitat. For example, *Microtus arvalis* is assigned to 0.5 open dry (OD) and 0.5 woodland (Wo) environments in Belgium [63] and 0.75 OD and 0.25 Wo in Italy [64]. In our current study, the *M*. *arvalis/agrestis* group weighting is divided over three habitat types (Table 2), as we take into consideration a larger geographic range (which includes most of Central Europe with Austria, Croatia, the Czech Republic, Germany, Hungary, Poland, Slovenia, Switzerland, Belgium, the Netherlands, and Luxembourg). Dividing the weighting of a taxon across multiple habitat types allows the addition of species previously considered as ‘indeterminate’ indicator species, such as the *Microtus arvalis/agrestis* group. This flexibility also allows the inclusion of family and genus level, or higher, identifications by dividing the weighting across all habitat types populated by the species within the group [27]. For the current study, this was done at the family and genus level for the single Muridae and Gliridae specimens and the *Myotis* sp., *Talpa* sp., *Sorex* sp., and *Crocidura* sp. individuals. For example, the *Sorex* sp. specimens were weighted with 0.75 woodland environments as *S*. *minutus*, *S*. *magna*, *S*. *pinus* and *S*. *coronatus* occupy woodlands more commonly than *S*. *anomalus*. Mitchel-Jones et al. [58] was used to determine the habitat distributions of extant species. We estimated the habitat weighing of species currently extinct or extirpated within Central Europe (which includes *Microtus gregalis* and *Spermophilus superciliosus*) from Kurten [60] and van Kolfschoten [1]. A list of the taxa used in this method and their habitat type weighing can be found in Table 2.

**Table 2:** List of species identified at Hohle Fels Cave and their weighted habitat preferences.

Species	O. Dry	O. Humid	O. Wood	Woodland	Rocky	Water	Steppe	Tundra
Chiroptera indet.					1			
*Myotis* sp.			0.2	0.4		0.4		
*Talpa* sp.		0.4	0.5				0.1	
Crocidura sp.	0.3	0.4					0.3	
*Crocidura leucodon/russula*		0.5					0.5	
*Crocidura leucodon*	0.25	0.75						
*Sorex* sp.		0.25		0.75				
*Sorex* cf. *araneus*		0.25		0.50		0.25		
*Sorex araneus*		0.25		0.50		0.25		
*Neomys* cf. *anomalus*						1		
*Neomys fodiens*		0.25				0.75		
*Spermophilus superciliosus*							1	
*Spermophilus* sp.			0.3				0.7	
Muridae			0.6	0.2			0.2	
Gliridae	0.15			0.85				
*Arvicola terrestris/antiquus*						1		
*Dicrostonyx gulielmi*								1
*Lemmus lemmus*								1
*Microtus gregalis*			0.25					0.75
*Chionomys nivalis*					1			
*Microtus oeconomus*	0.25	0.5				0.25		
*Microtus subterraneus*	0.25	0.25	0.25	0.25				
*Microtus arvalis/agrestis*	0.25	0.25	0.25	0.25				

## Results

### General taphonomy

We documented the presence and intensity of nine different taphonomic modifications within the Hohle Fels assemblage. Seven of these modifications are detailed in Table 3 and include evidence of discolouration from burning, the adherence of authigenic oxide minerals to the bone/tooth surface, occurrences of bone or enamel cracking leading to either perpendicular (even) or jagged (uneven) break edges, rounding of the bone surface and broken edges due to fluvial or sedimentary tumbling, tunneling of the bone surface due to the adherence of mineral seeking plant roots, and weathering of the bone surface due to exposure to wind, rain, and/or sunlight for extended periods.

**Table 3:** Skeletal element representation as expected in one small mammal individual (E) and by geological horizon (GH) and the tally of identified taphonomic modifications from Hohle Fels Cave.

Geological Horizon (GH)		GH 7		GH 7a/7aa		GH 8		GH 9		GH 10		GH 11		GH 12		Grand Total	
Skeletal Element	E	NSP	%	NSP	%	NSP	%	NSP	%	NSP	%	NSP	%	NSP	%	NSP	%
Mandible	2	25	10.2%	25.00	3.3%	116.00	5.7%	123	3.5%	10.00	2.1%	73.00	5.5%	464.00	5.1%	836	4.8%
Maxilla	2	2	0.8%	19.00	2.5%	49.00	2.4%	80	2.3%	9.00	1.8%	37.00	2.8%	253.00	2.8%	449	2.6%
Scapula	2	0	0.0%	10	1.3%	23	1.1%	51	1.4%	4	0.8%	8	0.6%	126	1.4%	222	1.3%
Humerus	2	24	9.8%	53	7.0%	158	7.7%	227	6.4%	35	7.2%	106	8.0%	677	7.4%	1280	7.3%
Radius	2	4	1.6%	8	1.1%	30	1.5%	93	2.6%	8	1.6%	39	3.0%	204	2.2%	386	2.2%
Ulna	2	10	4.1%	25	3.3%	73	3.6%	150	4.2%	31	6.4%	45	3.4%	385	4.2%	719	4.1%
Pelvis	2	15	6.1%	27	3.6%	57	2.8%	105	3.0%	10	2.1%	36	2.7%	330	3.6%	580	3.3%
Femur	2	32	13.1%	58	7.7%	195	9.5%	224	6.3%	31	6.4%	76	5.8%	682	7.5%	1298	7.4%
Tibia	2	29	11.8%	70	9.3%	183	9.0%	284	8.0%	67	13.8%	143	10.8%	823	9.0%	1599	9.1%
Vertebra	36	4	1.6%	25	3.3%	47	2.3%	161	4.5%	9	1.8%	53	4.0%	477	5.2%	776	4.4%
Incisor	4	38	15.5%	125	16.5%	322	15.8%	440	12.4%	65	13.3%	188	14.3%	1142	12.5%	2320	13.2%
Molar	12	38	15.5%	215	28.4%	559	27.3%	897	25.3%	114	23.4%	318	24.1%	2457	26.9%	4598	26.2%
Premolar	-	1	0.4%	1	0.1%	9	0.4%	7	0.2%	1	0.2%	8	0.6%	27	0.3%	55	0.3%
Astragalus/calcaneus	4	4	1.6%	8	1.1%	16	0.8%	51	1.4%	4	0.8%	10	0.8%	110	1.2%	203	1.2%
Rib	24	1	0.4%	14	1.9%	35	1.7%	112	3.2%	12	2.5%	13	1.0%	166	1.8%	353	2.0%
Metapodial	20	10	4.1%	63	8.3%	157	7.7%	478	13.5%	66	13.6%	146	11.1%	698	7.6%	1618	9.2%
Phalanx	56	8	3.3%	10	1.3%	15	0.7%	69	1.9%	11	2.3%	19	1.4%	122	1.3%	254	1.4%
Grand Total	174	245	100.0%	756	100.0%	2044	100.0%	3552	100.0%	487	100.0%	1318	100.0%	9143	100.0%	17546	100.0%
MNI	1																
Burning		20	12.4%	18	11.2%	39	24.2%	26	16.1%	23	14.3%	18	11.2%	15	9.3%	161	0.9%
Oxide		207	1.4%	718	4.7%	1711	11.2%	3306	21.6%	457	3.0%	1260	8.2%	7640	49.9%	15304	87.2%
Cracking (Uneven)		2	16.7%	2	16.7%	5	41.7%	1	8.3%	5	41.7%	3	25.0%	3	25.0%	12	0.1%
Cracking (Even)		2	1.4%	9	6.4%	42	30.0%	49	35.0%	18	12.9%	11	9.0%	9	6.4%	140	0.8%
Rounding		6	22.2%	4	14.8%	11	40.7%	1	3.7%	5	18.5%	9	33.3%	9	33.3%	27	0.2%
Root etching		2	11.8%	0	0.0%	3	17.6%	1	5.9%	0	0.0%	2	11.8%	9	52.9%	17	0.1%
Weathering		0	0.0%	2	50.0%	0	0.0%	1	25.0%	1	25.0%	0	0.0%	0	0.0%	4	0.0%
Juvenile specimens		5	1.5%	10	3.0%	2	0.6%	54	16.3%	9	2.7%	29	8.8%	222	67.1%	331	1.9%

With the exception of oxide mineral discolouration, all of these taphonomic agents had a very limited effect on the Hohle Fels small mammal sample. The highest proportion of discoloured specimens due to exposure to high or long duration temperatures occurs in GH 7 where 8.2% of all specimens were discoloured. Within all assemblages only 17 specimens were identified to Stiner’s ‘lightly burnt’ code 2. These specimens are also distributed throughout the stratigraphic sequence without any clear temporal relationship. There were no specimens recorded with burning beyond Stiner’s code 2. This is surprising considering that GH 7a/7aa contains a number of potentially anthropogenic hearth features. Rhodes et al. [65] have demonstrated that thermal discolouration of small mammal remains can accompany *in situ* combustion events within prehistoric deposits, and therefore a higher proportion of thermally modified specimens would be expected from within the GH 7a/7aa assemblage. As the degree of potential thermal discolouration throughout all assemblages did not warrant examination using more advanced methods, such as scanning electron microscopy or fourier-transform infrared spectroscopy, it remains possible that these specimens are in actuality exhibiting a form of anomalous oxide staining, as these two types of bone discolouration can be quite similar under visual examination [66,67].

The authigenic oxide staining prevalent throughout the Hohle Fels small mammal assemblage was differentiated from thermal discolouration visually based on its characteristic dendritic patterning, exclusive black colour, and metallic shine under high magnification. However, of the 85.7% of the small mammal sample with oxide discolouration, 3.9% had a staining morphology more appropriately described as a ‘wash,’ in which large continuous portions of the bone or tooth surface were discoloured to a light grey-black colour like that described by Marín-Arroyo et al. [51]. This oxide wash occurs on samples spanning the entire depth of the archaeological sequence included in this study, which suggests the depositional context producing this staining occurred during multiple temporal periods. It is possible that this anomalous staining indicates areas of increased moisture content within the sedimentary matrix or periods of water-logging during which the bones may have soaked in mineral rich water allowing a greater portion of the bone surface to become homogenously discoloured. The majority of specimens exhibiting both dendritic and ‘wash’ oxide staining exhibited the discolouration over <10% of the bone surface, and those with heavier deposition, either in characteristic dendritic patterning or as a wash, show no clear vertical patterning throughout the depth of deposits.

Although we documented rounding of element break edges on 2.5% of the specimens from GH 7, this modification was found on far fewer specimens throughout the rest of the assemblage (0.1% total). This suggests that despite the potentially high sedimentary moisture content indicated by the oxide staining, the assemblage was not subject to tumbling or sediment abrasion by water movement either pre- or post-depositionally. The high proportion of rounded specimens in GH 7 is more likely a result of the small sample size in this horizon than of any unique accumulation events. The other taphonomic modifications detailed in Table 1 occur in such small amounts as to not warrant further discussion.

The limited evidence of rounding, as well as the lack of articulated specimens and the heterogeneous nature of the taxonomic composition of the assemblage (see below) eliminate fluvial transport and mass death as possible mechanisms of accumulation for the assemblage. Instead, predation by owls, diurnal raptors, or mammalian carnivores remain the most likely methods by which the assemblage was deposited. To determine which small mammal predators were active at the site through time we conducted detailed analyses of the skeletal element representation and fragmentation, and the degree of digestive corrosion on the dental and select post-cranial elements.

### Predation related modifications

#### Skeletal element representation and breakage

We present skeletal element representation in Table 3 and the breakage patterns of certain post-cranial and cranial elements in Table 4 and Table 5, both of which are comparable to actualistic [48] and other archaeological [18,49,54,68–71] studies. However, it is important to be cautious when using skeletal element representation and/or breakage as indicators of assemblage accumulation since studies of small mammal bone density-mediated attrition are rare [54], and generally utilize density measurements of single species [72, 73] making comparison with multi-species assemblages less than ideal [54]. It has also been shown that post-depositional trampling can mimic patterns of bone breakage and loss seen in assemblages produced by moderately destructive predators [18,48]. The high site occupation intensity suggested by the artifact densities of the Aurignacian horizons at Hohle Fels [74], would imply that material deposited before and during the Aurignacian period may have been subject to substantial post-depositional trampling breakage. However, fragmentation data from these horizons are still useful as they can corroborate other lines of evidence related to assemblage accumulation (i.e. digestive etching).

**Table 4:** Number (NSP) and proportion (%) of long bones by geological horizon at Hohle Fels Cave.

Geological Horizon (GH)	GH 7		GH 7a/7aa		GH 8		GH 9		GH 10		GH 11		GH 12		Grand Total	
	NSP	%	NSP	%	NSP	%	NSP	%	NSP	%	NSP	%	NSP	%	NSP	%
Femur																
Complete	3	9.3%	5	8.8%	4	2.1%	16	7.4%	1	3.2%	10	13.3%	66	9.8%	105	8.2%
Proximal	17	53.1%	31	54.4%	94	48.4%	101	46.8%	23	74.2%	34	45.3%	345	51.1%	645	50.4%
Shaft	9	28.1%	13	22.8%	74	38.1%	63	29.2%	6	19.4%	20	26.7%	129	19.1%	314	24.5%
Distal	3	9.4%	8	14.0%	22	11.3%	36	16.7%	1	3.2%	11	14.7%	135	20.0%	216	16.9%
Total	32	100.0%	57	100.0%	194	100.0%	216	100.0%	31	100.0%	75	100.0%	675	100.0%	1280	100.0%
*Humerus*																
Complete	2	8.3%	5	9.6%	2	1.3%	18	8.0%	1	2.9%	12	11.3%	101	14.9%	141	11.1%
Proximal	0	0.0%	6	11.5%	14	9.4%	36	15.9%	1	2.9%	17	16.0%	167	24.7%	241	19.0%
Shaft	0	0.0%	7	13.5%	22	14.8%	45	19.9%	13	37.1%	18	17.0%	77	11.4%	182	14.4%
Distal	22	91.7%	34	65.4%	111	74.5%	127	56.2%	20	57.1%	59	55.7%	331	49.0%	704	55.5%
Total	24	100.0%	52	100.0%	149	100.0%	226	100.0%	35	100.0%	106	100.0%	676	100.0%	1268	100.0%
*Tibia*																
Complete	3	10.0%	0	0.0%	1	0.5%	2	0.7%	2	3.0%	3	2.1%	6	0.7%	17	1.1%
Proximal	7	23.3%	14	20.0%	47	25.5%	78	27.5%	15	22.4%	32	22.7%	287	35.7%	480	30.4%
Shaft	9	30.0%	20	28.6%	62	33.7%	91	32.0%	25	37.3%	50	35.5%	234	29.1%	491	31.1%
Distal	11	36.7%	36	51.4%	74	40.2%	113	39.8%	25	37.3%	56	39.7%	276	34.4%	591	37.4%
Total	30	100.0%	70	100.0%	184	100.0%	284	100.0%	67	100.0%	141	100.0%	803	100.0%	1579	100.0%
*Ulna*																
Complete	0	0.0%	2	7.1%	5	6.9%	14	9.2%	0	0.0%	3	6.7%	34	8.8%	58	8.0%
Proximal	10	100.0%	12	42.9%	41	56.9%	82	53.9%	16	51.6%	26	57.8%	191	49.6%	378	52.3%
Shaft	0	0.0%	14	50.0%	24	33.3%	53	34.9%	11	35.5%	14	31.1%	110	28.6%	226	31.3%
Distal	0	0.0%	0	0.0%	2	2.8%	3	2.0%	4	12.9%	2	4.4%	50	13.0%	61	8.4%
Total	10	100.0%	28	100.0%	72	100.0%	152	100.0%	31	100.0%	45	100.0%	385	100.0%	723	100.0%
*Total*																
Complete	8	8.3%	12	5.8%	12	2.0%	50	5.7%	4	2.4%	28	7.6%	207	8.2%	321	6.6%
Proximal	34	35.4%	63	30.4%	196	32.7%	297	33.8%	55	33.5%	109	29.7%	990	39.0%	1744	36.0%
Shaft	18	18.8%	54	26.1%	182	30.4%	252	28.7%	55	33.5%	102	27.8%	550	21.7%	1213	25.0%
Distal	36	37.5%	78	37.7%	209	34.9%	279	31.8%	50	30.5%	128	34.9%	792	31.2%	1572	32.4%
Total	96	100.0%	207	100.0%	599	100.0%	878	100.0%	164	100.0%	367	100.0%	2539	100.0%	4850	100.0%

**Table 5:** Number (NSP) and proportion (%) of maxillae and mandibles including proportion of specimens exhibiting various breakage categories and comparative breakage indices (both following Andrew, 1990) from Hohle Fels Cave. See the text for interpretation.

	GH 7	GH 7a/7aa	GH 8	GH 9	GH 10	GH 11	GH 12
Total Maxillae (NSP)	2	19	49	80	9	37	253
% complete	0.0%	0.0%	0.0%	0.0%	0.0%	0.0%	0.0%
% maxillae with zygomatic	50.0%	0.0%	12.2%	17.5%	22.2%	24.3%	11.4%
% palates	50.0%	100.0%	87.8%	82.5%	77.8%	75.7%	88.6%
Total Mandibles (NSP)	25	25	116	123	10	73	464
% complete	0.0%	0.0%	0.0%	0.8%	2.6%	0.0%	3.0%
% anterior break	68.0%	60.0%	67.2%	82.9%	70.0%	43.8%	29.7%
% inferior border break	40.0%	60.0%	75.9%	67.5%	40.0%	64.4%	30.8%
% ramus break	68.0%	84.0%	91.4%	92.7%	80.0%	86.3%	86.0%
Breakage Index							
post-crania/crania	221.1	122.1	131.0	165.1	171.5	158.9	153.3
femur+humerus/mandible+maxilla	207.4	252.3	213.9	222.2	347.4	165.5	189.5
tibia+radius/femur+humerus	58.9	70.3	60.3	83.6	113.6	100.0	75.6
% isolated molars*	38.8%	115.1%	85.9%	105.6%	137.0%	70.1%	83.0%
% molar occlusal break	18.4%	18.1%	9.8%	12.3%	16.6%	16.9%	12.3%

*calculated by dividing the # isolated molars by the # expected molars (based on 3 per jaw) minus the in situ molars

**Fig 3:**
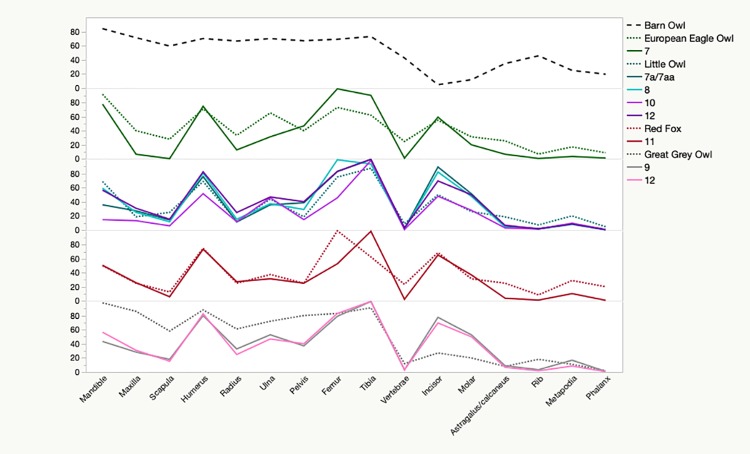
Skeletal element relative abundance (R) by geological horizon at Hohle Fels Cave. Only horizons showing a strong correlation (τ = > 0.600) with modern prey samples (from Andrews, 1990) are included. We include the pattern produced by barn owls (*Tyto alba*) at the top for comparison, as this low modifying predator approximates the pattern expected if perfect preservation of all elements were to occur.

At Hohle Fels the small mammal skeletal element representation is surprisingly uniform through time. Although the sample size between geological horizons varies greatly (from 244 to 9116 specimens), the proportional value of each element is rather homogeneous throughout all deposits, as well as when the assemblages are considered as a whole (Table 3). When the raw data are converted into relative proportions, the overall pattern can be compared to those recovered from actualistic assemblages both visually (Fig 3) and statistically (Table 6). We identified strong correlations (τ >6.000; Table 6 and Fig 3) between the patterns produced by the little owl (*Athena noctua*), red fox (*Vulpes vulpes*), great grey owl (*Strix nebulosa*), and European eagle owl (*Bubo bubo*) and those identified in the Hohle Fels element abundances from horizons 7 through 12. The ‘zig-zag’ pattern of the relative abundance seen in Fig 3 indicates preferential preservation of higher density elements [48], specifically the humerus, femur, tibia, and, to a lesser extent, the ulna. The relative amount of isolated dental elements (incisors and molars) is also quite high, ranging from 20% to 80%, (Fig 3) and this is reflected in the low number of maxillae and mandibles present. At the nearby site of Geißenklösterle, archaeological skeletal element abundances were also strongly correlated with the patterns produced by the European eagle owls, little owls, red foxes, and kestrels (*Falco tinnunculus*), however the relative proportion of isolated dentition was higher (50–100%), suggesting that comparatively the Hohle Fels material was subject to less intensive fragmentation. Few complete specimens were recovered of either post-crania (Table 4) or cranial elements (Table 5), suggesting that none of the assemblages were accumulated by low-modifying owls or raptors. Comparison of the different long bone breakage classes (Table 4) with Andrews’ [48] actualistic data suggests that mammalian predators, particularly the red fox, or heavily modifying birds of prey such as the little owl and hen harrier may be responsible for accumulating all the Hohle Fels material.

**Table 6:** Kendall’s Tau (τ) correlation results by geological horizon at Hohle Fels Cave showing all significant correlations > 0.600.

Geological Horizon (GH)	Actualistic predator pattern (adapted from Andrews, 1990)	*τ*	P score (<0.05 significant)
7	European Eagle Owl	0.7745	<0.0001
	Red Fox	0.7392	0.0001
	Coyote	0.7009	0.0002
	Kestrel	0.6950	0.0002
	Little Owl	0.6466	0.0007
7a/7aa	Little Owl	0.7575	<0.0001
	Red Fox	0.6611	0.0005
	European Eagle Owl	0.6387	0.0006
8	Little Owl	0.7798	<0.0001
	Red Fox	0.7524	<0.0001
	European Eagle Owl	0.7280	<0.0001
	Kestrel	0.6500	0.0004
	Coyote	0.6219	0.0008
9	Little Owl	0.7628	<0.0001
	Red Fox	0.7182	0.0001
	European Eagle Owl	0.6778	0.0003
	Kestrel	0.6333	0.0006
	Coyote	0.6219	0.0008
	Great Grey Owl	0.6000	0.0012
10	Little Owl	0.7351	<0.0001
	Red Fox	0.7070	0.0002
	European Eagle Owl	0.6498	0.0005
	Kestrel	0.6051	0.0012
11	Red Fox	0.7524	<0.0001
	Little Owl	0.7459	<0.0001
	Coyote	0.6891	0.0002
	Kestrel	0.6667	0.0003
	European Eagle Owl	0.6611	0.0004
	Short-eared owl	0.6167	0.0009
	Hen Harrier	0.6051	0.0012
12	Little Owl	0.8306	< .0001
	Red Fox	0.7695	< .0001
	European Eagle Owl	0.7448	< .0001
	Kestrel	0.6667	0.0003
	Coyote	0.6387	0.0006
	Great Grey Owl	0.6000	0.0012

Similarly, the lack of complete crania in the assemblages and the prevalence of isolated palates and mandibular fragments (Table 5) attests to the high level of overall destruction, also suggesting a mammalian predator as the main accumulator. In geological horizons 10 and 11 the proportion of maxillae and zygomatic specimens reaches similar levels to those seen in short-eared owl assemblages, a predator which was also suggested by the post-cranial skeletal element representation of GH 11 (Table 3). The number of complete mandibles is likewise low, totaling less than 7% of the total assemblage and found only in the MP deposits (GH 9–12). The percent of specimens exhibiting broken rami and/or a break along the inferior border of the corpus falls within ranges produced by the kestrel, hen harrier, mongoose, and other mammalian predators according to Andrews [48: Table 3.7] for all horizons. The high degree of mandibular and maxillae breakage is further indicated in the proportion of isolated molars, presented here as an index which is calculated by dividing the number of isolated molars by the total molars expected based on empty alveolar spaces present in the recovered mandibles and maxillae (Table 5). The results again fall within the range produced by the kestrel, hen harrier, mongoose, and other mammalian predators in geological horizons 7, 8, 11, and 12, whereas results higher than 100%, as in horizons 7a/7aa, 9, and 10, indicate the complete loss of mandibular elements.

#### Digestive corrosion on dental elements

Due to the distinct possibility that post-depositional trampling and sediment compression has increased the level of skeletal element breakage, we also documented the presence and degree of digestive corrosion on incisors (Table 7), molars (Table 8 and Table 9) and select post-cranial elements (Table 10 and Table 11). Corrosion due to exposure to digestive enzymes is unique in that it can be differentiated from all processes which produce similar alteration of bones and teeth through careful examination of the morphology and extent of the modification.

**Table 7:** Number (N) and proportion (%) of digested incisors out of all incisors by geological horizon divided by area of modification from Hohle Fels Cave.

Geological Horizon (GH)	Tip Digestion												Total Tip Digested	
	Absent		Light		Light-Moderate		Moderate		Moderate-Heavy		Heavy			
	NSP	%	NSP	%	NSP	%	NSP	%	NSP	%	NSP	%	NSP	%
7	28	73.7%	3	7.9%		0.0%	2	5.3%	1	2.6%		0.0%	6	15.8%
7a/7aa	101	80.8%	6	4.8%	2	1.6%		0.0%		0.0%	1	0.8%	9	7.2%
8	269	83.5%	5	1.5%	3	0.9%		0.0%		0.0%		0.0%	8	2.4%
9	387	88.0%	10	2.3%		0.0%		0.0%		0.0%		0.0%	10	2.3%
10	58	89.2%	3	4.6%	2	3.1%		0.0%		0.0%		0.0%	5	7.7%
11	170	89.9%	4	2.1%	2	1.1%	1	0.5%	1	0.5%		0.0%	8	4.2%
12	1003	87.9%	62	5.4%	4	0.4%	2	0.2%	1	0.1%	1	0.1%	70	6.1%
Grand Total	2016	86.9%	93	4.0%	13	0.6%	5	0.2%	3	0.1%	2	0.1%	116	5.0%
Geological Horizon (GH)	Surface Digestion								Total Surface Digested		Grand Total	
	Light		Light-Moderate		Moderate		Moderate-Heavy					
	NSP	%	NSP	%	NSP	%	NSP	%	NSP	%	NSP	%
7	1	0.0%	2	0.0%	1	2.6%		0.0%	4	10.5%	38	1.6%
7a/7aa	12	9.6%	3	2.4%		0.0%		0.0%	15	12.0%	125	5.4%
8	25	7.8%	19	5.9%	1	0.3%		0.0%	45	13.9%	322	13.9%
9	22	5.0%	16	3.6%	3	0.7%	2	0.5%	43	9.7%	440	19.0%
10	2	3.0%		0.0%		0.0%		0.0%	2	3.0%	65	2.8%
11	8	4.2%	3	1.6%		0.0%		0.0%	11	5.8%	189	8.1%
12	51	4.4%	11	0.9%	5	0.4%	1	0.1%	68	5.9%	1141	49.2%
Grand Total	121	5.2%	54	2.3%	10	0.4%	3	0.1%	188	8.1%	2320	100.0%

**Table 8:** Digestion of isolated Arvicolid dental elements by geological horizon and etching intensity from Hohle Fels Cave. Includes all molars to allow comparability with Andrews 1990.

Geological Horizon (GH)	*Lemmus lemmus*	*Dicrostonyx gulielmi*	*Microtus gregalis*	*Microtus oeconomus*	*Chionomys nivalis*	*Arvicola terrestris/antiquus*	*Microtus subterraneous*	*Microtus arvalis/agrestis*	*Microtus* sp.	Total	%
7	4	2	0	5	0	0	0	8	30	49	
absent	4	2		5				6	30	47	95.9%
light								2		2	4.1%
7a/7aa	34	10	13	9	3	4	0	34	133	240	
absent	31	10	9	7	2	4		26	119	208	86.7%
Light	2		3	2	1			5	10	23	9.6%
Light-moderate								2		2	0.8%
Moderate	1		1					1	4	7	2.9%
8	72	22	36	8	4	5	0	92	421	660	
absent	65	19	30	7	3	5		82	412	623	94.4%
light	5	2	5	1				5	7	25	3.8%
Light-moderate	1		1					1		3	0.5%
Moderate	1	1			1			3	2	8	1.2%
Heavy								1		1	0.2%
9	73	14	48	15	2	5	0	123	761	1041	
absent	65	14	42	12	2	4		101	759	999	96.0%
Light	3		5			1		16	1	26	2.5%
Light-moderate								1		1	0.1%
Moderate	1		1	1				3		6	0.6%
Moderate—heavy	2			2						4	0.4%
Heavy	2							2		4	0.4%
Extreme									1	1	0.1%
10	6	2	3	3	0	1	0	16	95	126	
absent	6	2	2	2		1		12	91	116	92.1%
Light			1	1				4	2	8	6.3%
Moderate									2	2	1.6%
11	19	5	5	11	8	18	0	74	243	383	
absent	18	5	4	9	8	18		57	243	362	94.5%
light	1		1	2				13		17	4.4%
Light-moderate								2		2	0.5%
moderate								2		2	0.5%
12	261	40	90	31	7	13	2	397	2040	2881	
absent	243	38	75	22	6	13	1	321	2040	2759	95.8%
light	4		9	7			1	56		77	2.7%
Light-moderate			1					8		9	0.3%
moderate	11	1	3	1	1			5		22	0.8%
Moderate—heavy	1		1	1				2		5	0.2%
Heavy	1	1						2		4	0.1%
Heavy-extreme	1		1					3		5	0.2%
Isolated total	469	95	195	82	24	46	2	744	3723	5380	
absent total	432	90	162	64	21	45	1	605	3694	5114	95.1%
light total	15	2	24	13	1	1	1	101	20	178	3.3%
Light—Moderate total	1	0	2	0	0	0	0	14	0	17	0.3%
Moderate total	14	2	5	2	2	0	0	14	8	47	0.9%
Moderate—Heavy total	3	0	1	3	0	0	0	2	0	9	0.2%
Heavy total	3	1	0	0	0	0	0	5	0	9	0.2%
Heavy—Extreme total	1	0	1	0	0	0	0	3	0	5	0.1%
Extreme total	0	0	0	0	0	0	0	0	1	1	0.0%
%absent	92.1%	94.7%	83.1%	78.0%	87.5%	97.8%	50.0%	81.3%	99.2%	95.1%	
%light	3.2%	2.1%	12.3%	15.9%	4.2%	2.2%	50.0%	13.6%	0.5%	3.3%	
%light—moderate	0.2%	0.0%	1.0%	0.0%	0.0%	0.0%	0.0%	1.9%	0.0%	0.3%	
%moderate	3.0%	2.1%	2.6%	2.4%	8.3%	0.0%	0.0%	1.9%	0.2%	0.9%	
%moderate—heavy	0.6%	0.0%	0.5%	3.7%	0.0%	0.0%	0.0%	0.3%	0.0%	0.2%	
%heavy	0.6%	1.1%	0.0%	0.0%	0.0%	0.0%	0.0%	0.7%	0.0%	0.2%	
%heavy-extreme	0.2%	0.0%	0.5%	0.0%	0.0%	0.0%	0.0%	0.4%	0.0%	0.1%	
%extreme	0.0%	0.0%	0.0%	0.0%	0.0%	0.0%	0.0%	0.0%	0.0%	0.0%	

**Table 9:** Digestion of in situ Arvicolid dental elements by geological horizon and etching intensity from Hohle Fels Cave. Includes all molars to allow comparability with Andrews, 1990.

Geological Horizon (GH)	*Lemmus lemmus*	*Dicrostonyx gulielmi*	*Microtus gregalis*	*Microtus oeconomus*	*Chionomys nivalis*	*Arvicola terrestris/antiquus*	*Microtus arvalis/agrestis*	*Microtus* sp.	Total	%
7a/7aa	2		2		2		5	6	17	
Absent	2		2		2		4	6	16	94.1%
Light									0	0.0%
Moderate							1		1	5.9%
8	4	4	9		3	2	26	17	65	
Absent	4	4	9		3	2	24	16	62	95.4%
Light							2	1	3	4.6%
Moderate									0	0.0%
9	3		11	2	3		6	17	42	
Absent	3		11	2	3		6	17	42	100.0%
11			3				7	14	24	
Absent			3				7	13	23	95.8%
Light								1	1	4.2%
12	46	13	43	3	2	4	108	98	317	
Absent	46	12	37	3	2	4	97	92	293	92.4%
Light			5				9	5	19	6.0%
Light—Moderate							2	1	3	0.9%
Moderate		1	1						2	0.6%
In situ total	55	17	68		10	6	152	152	465	
Absent total	55	16	62		10	6	138	144	436	
Light total	0	0	5		0	0	11	7	23	
Light—Moderate total	0	0	0		0	0	2	1	3	
Moderate total	0	1	1		0	0	1	0	3	
%absent	100.0%	94.1%	91.2%		100.0%	100.0%	90.8%	94.7%	93.8%	
%light	0.0%	0.0%	7.4%		0.0%	0.0%	7.2%	4.6%	4.9%	
%light—moderate	0.0%	0.0%	0.0%		0.0%	0.0%	1.3%	0.7%	0.6%	
%moderate	0.0%	5.9%	1.5%		0.0%	0.0%	0.7%	0.0%	0.6%	

**Table 10:** Number and percentage of proximal femurs exhibiting digestive corrosion damage by geological horizon from Hohle Fels cave.

Geological Horizon (GH)	Proximal Femur Digestion								Total Femur Digestion	
	Absent		Light		Light—Moderate		Moderate			
	NSP	%	NSP	%	NSP	%	NSP	%	NSP	%
7	8	40.0%	9	45.0%	1	5.0%	2	10.0%	12	60.0%
7a/7aa	28	77.8%	7	19.4%	1	2.8%		0.0%	8	22.2%
8	85	86.7%	11	11.2%	1	1.0%	1	1.0%	13	13.3%
9	91	77.8%	17	14.5%	7	6.0%	2	1.7%	26	22.2%
10	9	37.5%	7	29.2%	7	29.2%	1	4.2%	15	62.5%
11	27	61.4%	11	25.0%	2	4.5%	4	9.1%	17	38.6%
12	350	85.2%	17	4.1%	18	4.4%	26	6.3%	61	14.8%
Grand Total	598	79.7%	79	10.5%	37	4.9%	36	4.8%	152	20.3%

**Table 11:** Number and percentage of distal humeri exhibiting digestive corrosion by geological horizon from Hohle Fels cave.

Geological Horizon (GH)	Distal Humerus Digestion								Total Humerus Digestion	
	Absent		Light		Light—Moderate		Moderate			
	N	%	N	%	N	%	N	%	N	%
7	18	75.0%	6	25.0%		0.0%		0.0%	6	25.0%
7a/7aa	32	82.1%	7	17.9%		0.0%		0.0%	7	17.9%
8	106	93.8%	6	5.3%	1	0.9%		0.0%	7	6.2%
9	122	84.1%	15	10.3%	5	3.4%	3	2.1%	23	15.9%
10	14	66.7%	5	23.8%	2	9.5%		0.0%	7	33.3%
11	54	76.1%	12	16.9%	3	4.2%	2	2.8%	17	23.9%
12	403	93.3%	16	3.7%	12	2.8%	1	0.2%	29	6.7%
Grand Total	749	88.6%	67	7.9%	23	2.7%	6	0.7%	96	11.4%

**Fig 4:**
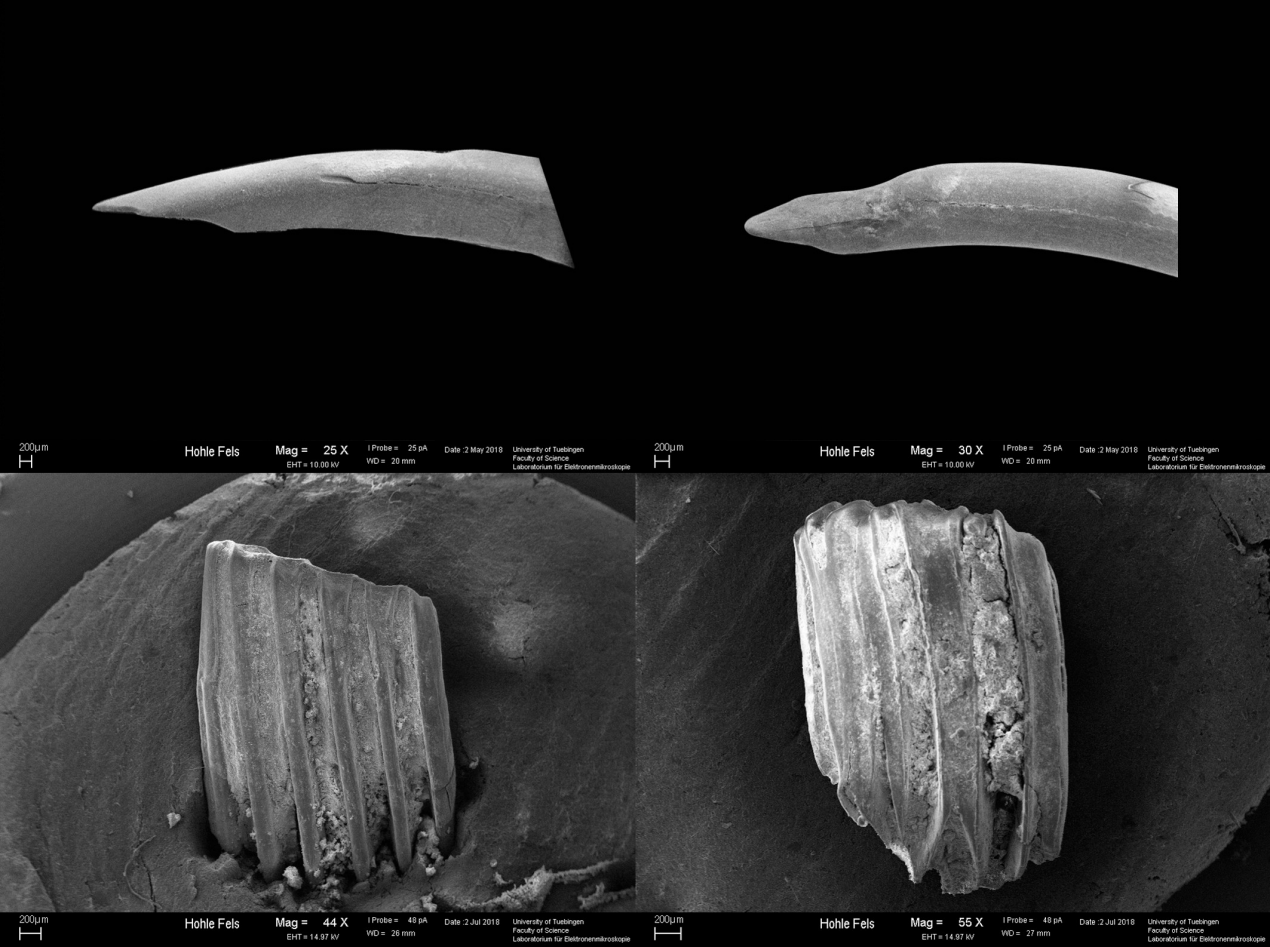
Scanning Electron Microscopy photos of digestive modification on dental elements from Hohle Fels Cave. Top left: lightly digested arvicolid incisor; Top right: moderately digested arvolid incisor; Bottom left: lightly digested arvicolid molar; Bottom right: heavily digested arvicolid molar.

Following Andrews [48], we documented digestion on both isolated and *in situ* incisors and present them here as combined totals (Table 5). The intensity of the modification on incisors is tallied along a continuous scale from light to extreme. Examples of light and moderate level digestion of incisors can be seen in Fig 4. Overall, the majority of incisors from Hohle Fels (73.7% to 89.9%) show no evidence of digestive corrosion and the highest proportions of specimens with either tip or surface corrosion exhibit only light retraction of the enamel or pockets of enamel loss. None of the specimens exhibit extreme digestion, which presents as a complete loss of the tooth enamel and heavy modification of the dentine core. Only one specimen from GH 7a/7aa and one from GH 12 was categorized as having heavy tip corrosion, in which the enamel was retracted posteriorly and the dentine was eroded and cracked across the body of the tooth. GH 9, and to a lesser extent GH 8, include the highest number of incisors with surface modification, which may indicate that more specimens from these layers were broken from their alveolar socket during predation, as isolated teeth experience greater surface acid exposure. The total amount of both tip and surface digestion fall within Andrews’ [48] digestion category 1 (Table 12), in which digestive modification is absent or minimal and is associated with barn owl, short-eared owl, and snowy owl (*Bubo scandiacus*) actualistic assemblages. Although the moderately damaged specimens might indicate the presence of more destructive predators, such as an eagle owl or little owl, there is no clear pattern in the distribution of these more heavily modified specimens across horizons to suggest a particular period of cave occupation by these predators.

**Table 12:** Categories of predators according to digestive modification (modified from Andrews, 1990).

Category	Predators	Alterations
1	*Molar digestion*: Barn, Long-eared, Short-eared owl, Verreaux eagle owl,	Light modification, absent or light digestion Molars: 0–3% Incisors: 8–13% Post-crania: 0–20%
	*Incisor digestion*: Barn owl, short-eared owl, snowy owls	
	*Post-cranial digestion*: Barn, snowy, long-eared, short-eared owls, Verreaux eagle owl, great grey owl	
2	*Molar digestion*: Snowy, spotted eagle, great grey owls	Little modification, moderate degree of digestion, though enamel is removed from the tips of incisors Molars: 4–6% Incisors: 20–30% Post-crania: 25–50%
	*Incisory digestion*: Long-eared owl, Verreaux eagle owl, great grey owl, bat-eared fox	
	*Post-cranial digestion*: European & spotted eagle owls, tawny owl	
3	*Molar digestion*: European eagle owl, tawny owl, bat-eared fox, mongoose, genet	Greater destruction, moderate/heavy digestion over the enamel Molars: 18–22% Incisors 50–70% Post-crania: 60–100%
	*Incisor digestion*: European & spotted eagle owls, tawny, little owl, pine marten, mongoose, genet	
	*Post-cranial digestion*: Little owl, kestrel, hen harrier, peregrine falcon	
4	*Molar digestion*: Little owl, kestrel, pine martin	Heavy/Extreme enamel and dentine corrosion; mustelids produce extreme modification but digested elements appear in low percentages, some of them chewed Molars: 50–70% Incisors: 60–80% Post-crania: ~100%
	*Incisor digestion*: Kestrel	
	*Post-cranial digestion*: same as category 5	
5	*Molar*, *incisor and post-cranial digestion*: Hen harrier, coyote, red fox, arctic fox, mammalian carnivores.	The most destructive effects (extreme). Mammalian carnivores produce rounded edges of skeletal elements. Gnaw marks rare, except for some instances of canid predation and of some mustelids Low percentages of digested post-crania and complete lack of cranio-dental elements. Molars: 50–100% Incisors: 100% (dentine corroded) Post-crania: ~100%

A similar pattern is seen when we consider the evidence for digestive corrosion on both isolated and *in situ* molars. The arvicolidae results are presented in Table 8 for isolated molars and Table 9 for *in situ* specimens. In actualistic predator assemblages the proportion of molars exhibiting digestive corrosion is significantly lower than the proportion of incisors [48]. At Hohle Fels, the majority of arvicolid molars have no evidence of digestive modification (86.7% - 96.0%) and only geological horizons 8, 9, and 12 include specimens with heavy to extreme modification (Fig 4). The remaining molars with light to moderate levels of digestive modification account for between 3.5% and 13.3% of the arvicolid teeth in any given horizon (Table 8 and Table 9). It is interesting to note that the specimens exhibiting greater than moderate modification come from two lemming species (*Lemmus lemmus* and *Dicrostonyx* sp.) and two vole species (*Microtus arvalis/agrestis* and *M*. *gregalis*). Identifying digestive etching on lemming molars is complicated by the natural lack of enamel along the salient edges of the teeth, which may cause low level modification to appear greater in intensity. This distinct tooth morphology also allows identification of all isolated lemming molars to genera level. Conversely, the vole dental pattern is more conservative with only the lower m1 exhibiting enough inter-species variation to allow taxonomic determination. As such, the lemming sample from Hohle Fels is inflated in relation to the vole sample, and the 4 specimens which exhibit heavy or extreme digestive corrosion may have originated from only 3 individuals, whereas the *M*. *arvalis/agrestis* and *M*. *gregalis* specimens adds up to at least 6 individuals. Still, the occurrence of digestive damage of greater intensity on just these four taxa from three horizons suggests that a diurnal raptor or mammalian predator with a dietary preference for voles and lemmings was active in the cave during these periods.

The overall proportion of digested isolated arvicolid molars from most geological horizons falls within Andrews’ [58] digestion category 2 and indicates the presence of a moderately destructive predator in the cave, such as a snowy owl, European eagle owl, or great grey owl. GH 7a/7aa and GH 10 have total counts that fall between Andrews’ category 2 and category 3, which may suggest that a European eagle owl or tawny owl was present when these deposits were accumulated. The proportion of modified *in situ* molars (Table 9) follows this trend, with most horizons having between 4.2% and 7.5% of the sample exhibit digestive corrosion. Two horizons, GH 7 and GH 10, had no *in situ* molars present, indicating a higher than expected loss of dentition and destruction of jaws. Overall, the proportion of *in situ* dentition ranges from 3.2% to 9.9% of all teeth examined, which suggests that retention of molars within their original alveolar sockets was low throughout all periods.

#### Digestive corrosion on post-cranial elements

**Fig 5:**
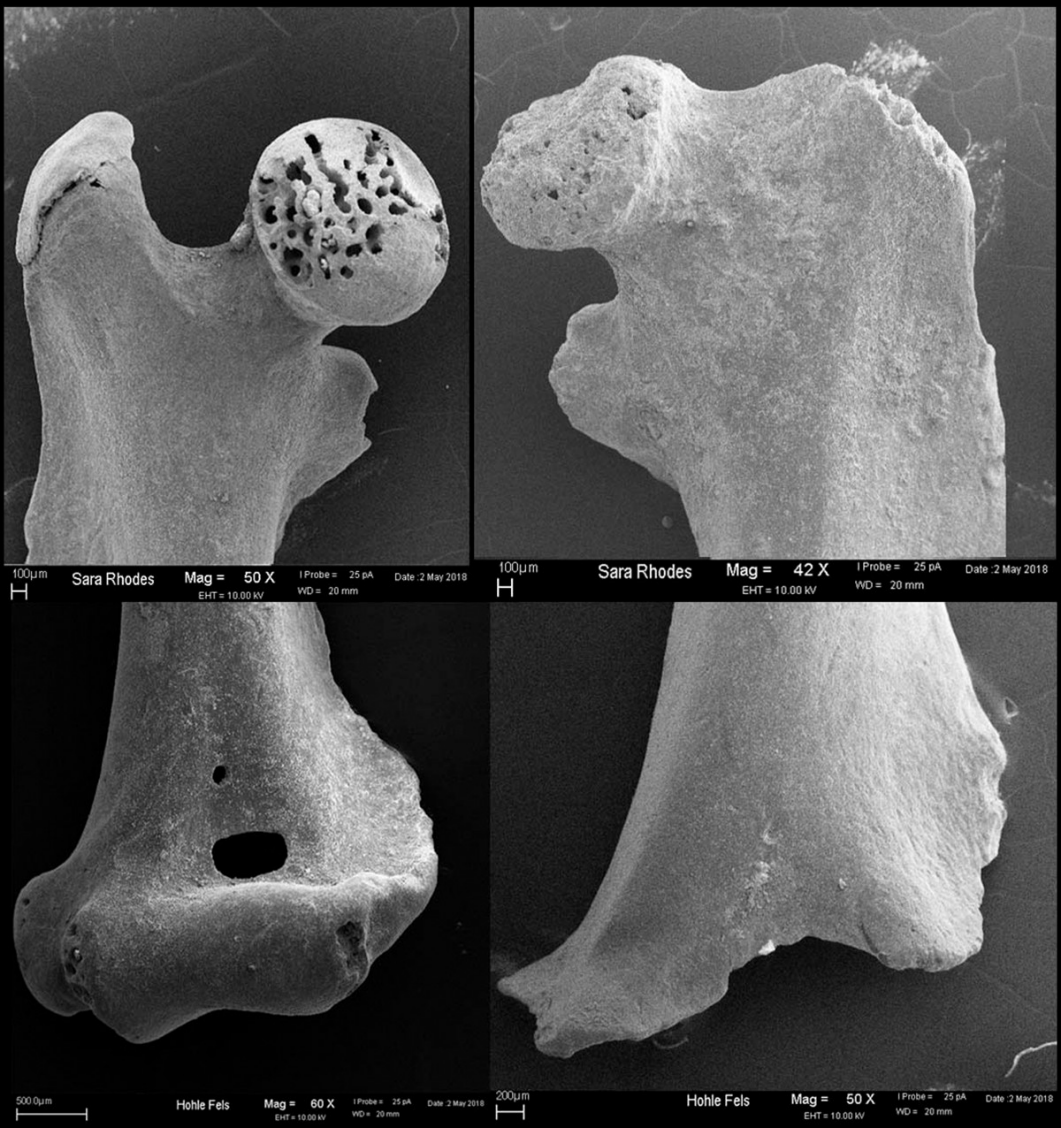
Scanning electron microscopy photographs of digestive modification on post-cranial specimens from Hohle Fels Cave. Top left: lightly digested proximal femur; Top right: heavily digested proximal femur; Bottom left: lightly digested distal humerus; Bottom right: heavily digested distal humerus.

At Hohle Fels, evidence of intrusive digestion is absent on most proximal femora and distal humeri with only 20.3% and 11.4% of all specimens, respectively, showing any level of corrosive modification (Table 10 and Table 11 and see Fig 5). When broken down by geological horizon, the range of digested femora varies from 13.3% to 62.5% throughout the deposits. The amount of digested distal humeri in each geological horizon falls within the range of 6.3% to 33.3%. Exactly why there exists such a discrepancy in the amount of digestion on humeri vs. femora is unclear, although it may relate to the speed of ossification and fusion between elements and species. Distal humeri have been shown to fuse at around three weeks of age in mice [75], whereas both the proximal and distal femur is ossified by the end of the fourth week [76] but only fused by the 13 – 15^th^ postnatal week [75]. Assuming voles and shrews follow a similar fusion pattern, late ossification and fusion rates may have increased the number of femora affected by digestive acid exposure in our sample. If this hypothesis is correct, then the distal humerus would prove a more reliable indicator of the category of accumulative predator than the proximal femur, and so when the two post-cranial records are in disagreement we give greater weight to the signal from the humerus. For example, in geological horizons 7, 7a/7aa, and 8 (the Aurignacian layers), the proportion of digested distal humeri fall within Andrews’ [58] low-modifying category 1 predators, including the barn owl, snowy owl, long-eared owl, and great grey owl; whereas the proportion of modified proximal femora suggest a category 2 or 3 predator was active when these deposits were laid down, such as the little owl, hen harrier, European eagle owl, spotted eagle owl or tawny owl. It is possible that predators from all three categories were active at the same time at the site. However, considering the signal for multiple predation patterns is coming from inter-bone comparisons, rather than inter-specific or relative abundance patterning, a more parsimonious explanation is that a low-modifying predator was active during all three periods and produced higher-than-expected levels of digestive corrosion on femoral heads due to the selective predation of juvenile individuals. The same logic can be applied to the contradictory signals within geological horizon 9, for which humeral proportions suggest a category 1 predator and femoral digestion suggests category 2 predator, and for geological horizon 10 which the post-cranial digestion data suggests may have been accumulated by category 3–5 predators such as the little owl or hen harrier, or by a category 2 predator such as the spotted or tawny owl. Only the two oldest MP horizons, geological horizon 11 and 12, showed agreement in all post-cranial corrosion and were likely accumulated by a category 2 predator (European eagle owl based on femoral digestion) and a category 1 predator, respectively.

#### Taphonomy: Summary and predator description

Table 13 summarizes the predators indicated by the various taphonomic indices used to evaluate the Hohle Fels small mammal assemblage. With few exceptions, the digestive evidence suggests that a category 1–2 predator was active throughout the sequence, while the skeletal element data suggests a category 4 or 5 predator was present in both the MP and Aurignacian. In all cases where the digestion and breakage results were not in agreement, we gave greater weight to the digestive evidence identified on isolated and *in situ* molars. This is most clearly seen with regards to GH 8, 9, and 12, the three largest subsamples of the Hohle Fels small mammal assemblage (Table 1). This also suggests that there may be a relationship between sample size and the presence of rare highly modified dental specimens. The predation signals from the other geological horizons all suggest that category 1–2 predators occupied the site. In the Aurignacian (GH 7 and 7a/7aa), this was likely a snowy owl, although the European eagle owl is also a possible accumulator, particularly in GH 7a/7aa. Regarding the MP, in GH 10 the eagle owl is also indicated in both the evidence of digestive corrosion and the correlation with actualistic skeletal element preservation. In GH 11 tooth digestion suggests either a great grey owl or snowy owl accumulated the small mammal material.

**Table 13:** Small mammal predators indicated by the different taphonomic indices applied to the Hohle Fels cave assemblage divided by geological horizon.

Geological Horizon (GH)	Barn owl	Short-eared owl	Snowy owl	Long-eared owl	Great grey owl	European eagle owl	Tawny owl	Little owl	Kestrel	Pine martin	Hen harrier	Golden Jackal	Red fox	Arctic fox	Modification type		
Predator Category	1,	1	1, 2	1,2	1,2	2,3	2,3	3,4	3,4	3,4	3,5	4,5	4,5	4,5			
7						x		x	x			x	x	x	Skeletal element representation		
								x			x		x	x	Skeletal element breakage		
									x	x	x	x	x	x	Cranial breakage		
		x				x						x			Post-crania to crania index		
													x	x	Femur+humerus/mand+max		
									x						Tibia+radius/femur+humerus		
									x	x	x	x	x	x	%isolated molars		
															% molar with occlusal breaks		
			x		x										Dig. Isolated molars		
												x	x	x	Dig. *In situ* molars		
	x	x	x												Dig. Incisors		
	x	x	x	x	x	x	x	x	x		x				Dig. Post-crania		
7a/7aa						x		x					x	x	Skeletal element representation		
								x			x		x	x	Skeletal element breakage		
									x	x	x	x	x	x	Cranial breakage		
															Post-crania to crania index		
													x	x	Femur+humerus/mand+max		
						x		x	x				x	x	Tibia+radius/femur+humerus		
												x	x	x	%isolated molars		
															% molar with occlusal breaks		
						x	x								Dig. Isolated molars		
			x		x										Dig. *In situ* molars		
	x	x	x												Dig. Incisors		
	x	x	x	x	x	x	x	x	x		x				Dig. Post-crania		
8						x		x	x			x	x	x	Skeletal element representation		
								x			x		x		Skeletal element breakage		
									x	x	x	x	x	x	Cranial breakage		
															Post-crania to crania index		
													x	x	Femur+humerus/mand+max		
											x				Tibia+radius/femur+humerus		
									x	x	x	x	x	x	%isolated molars		
											x				% molar with occlusal breaks		
					x								x**	x**	Dig. Isolated molars		
			x		x										Dig. *In situ* molars		
	x	x	x			x		x							Dig. Incisors		
	x	x	x	x	x	x	x	x	x		x				Dig. Post-crania		
9					x	x		x	x			x	x	x	Skeletal element representation		
								x			x		x	x	Skeletal element breakage		
									x	x	x	x	x	x	Cranial breakage		
															Post-crania to crania index		
													x	x	Femur+humerus/mand+max		
		x										x			Tibia+radius/femur+humerus		
												x	x	x	%isolated molars		
									x						% molar with occlusal breaks		
		x	x										x**	x**	Dig. Isolated molars		
			x		x										Dig. *In situ* molars		
						x		x							Dig. Incisors		
	x	x	x	x	x	x	x	x	x		x				Dig. Post-crania		
10						x		x	x				x	x	Skeletal element representation		
								x			x		x	x	Skeletal element breakage		
		x							x	x	x	x	x	x	Cranial breakage		
															Post-crania to crania index		
													x	x	Femur+humerus/mand+max		
	x														Tibia+radius/femur+humerus		
												x	x	x	%isolated molars		
								x							% molar with occlusal breaks		
						x									Dig. Isolated molars		
												x	x	x	Dig. *In situ* molars		
	x	x	x												Dig. Incisors		
						x	x	x			x				Dig. Post-crania		
11		x				x		x	x		x		x	x	Skeletal element representation		
								x			x		x	x	Skeletal element breakage		
		x							x	x	x	x	x	x	Cranial breakage		
															Post-crania to crania index		
								x							Femur+humerus/mand+max		
	x														Tibia+radius/femur+humerus		
									x	x	x	x	x	x	%isolated molars		
								x							% molar with occlusal breaks		
					x										Dig. Isolated molars		
			x		x										Dig. *In situ* molars		
	x	x	x												Dig. Incisors		
						**x**	x								Dig. Post-crania		
12					x	x		x	x			x	x	x	Skeletal element representation		
								x			x		x		Skeletal element breakage		
									x	x	x	x	x	x	Cranial breakage		
															Post-crania to crania index		
								x							Femur+humerus/mand+max		
						x			x				x	x	Tibia+radius/femur+humerus		
									x	x	x	x	x	x	%isolated molars		
									x						% molar with occlusal breaks		
		x	x										x**	x**	Dig. Isolated molars		
			x		x										Dig. *In situ* molars		
	x	x	x												Dig. Incisors		
	x	x	x	x											Dig. Post-crania		

Light grey tallies are based on comparison between taphonomic indices (i.e. lack of *in situ* dentition) or potentially anomalous data (i.e. % digested femoral heads).

** indicates <1% of sample exhibited predator modifications.

Our taphonomic results support the use of this assemblage for paleoenvironmental reconstruction as the European eagle owl, snowy owl, great grey owl, little owl, and both the red and arctic fox are all mainly non-selective hunters [48,58]. The snowy owl, European eagle owl and great grey owl are specialized hunters of arvicolids, specifically *Microtus*, *Lemmus* and *Dicrostonyx*, which comprise at least 50% of their diet [48,77] and which are taken on encounter and therefore reflect the relative abundance of the prey species on the landscape. However, snowy owl pellet assemblages have been shown to include lower than expected amounts of shew and murid taxa, and some studies suggest the great grey owl may select against squirrels and water voles and for shrews in their hunting behaviour [58]. As such, it is possible that more Soricids were present around Hohle Fels during the deposition of GH 7 and GH 9 than the taxonomic composition of these layers suggests. Furthermore, we may expect that GH 8 and GH 11 had a greater presence of grasslands and stream/lake beds around the site than our paleoenvironmental reconstruction indicates. The European eagle owl is known to preferentially hunt water voles, however this is clearly not the case in GH 7a/7aa or GH 10 where this species is found in very small numbers. The varied diet of the little owl, as well as its small size, make it unlikely to have been a major contributor to the Hohle Fels small mammal material [48,78]. However, like the red fox and arctic fox, the little owl hunts opportunistically and would therefore contribute a representative sample of the small mammal taxa present on the landscape surrounding its nest site. A slight selection against burrowing prey, such as the water vole, some murids, and moles can be expected in red fox assemblages, as would be a selection for lemmings by the arctic fox. When doing so will not result in some form of circular reasoning, as with the potential Arctic fox derived material, we also include the habitat preferences and/or requirements of the indicated predator species in our paleoenvironmental reconstruction as an additional source of vegetative and climatic data.

### Taxonomic composition

**Fig 6:**
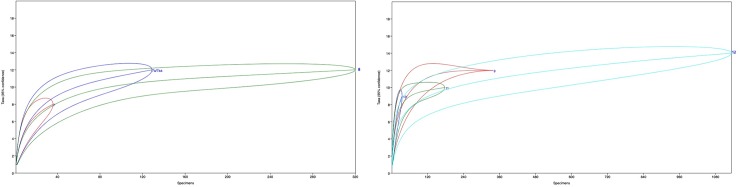
Rarefaction curves of species richness by geological horizon at Hohle Fels cave. Left) the Aurignacian geological horizons 7, 7a/7aa, and 8. Right) the Middle Paleolithic geological horizons 9, 10, 11 and 12.

The number of identified specimens (NISP) and minimum number of individuals (MNI) attributed to each taxonomic level are detailed in in Table 14. Seven taxonomic families, including 12 genera and 14 species were identified from the 6165 specimens comprising the Hohle Fels small mammal assemblage. Our rarefaction analysis suggests that the small sample sizes from geological horizons 7, 7a/7aa and 10 likely underrepresent rare taxa. Furthermore, a variation of between 2 and 3 taxonomic designations, at the 95% confidence level, is seen in all but the largest horizons (Fig 6).

**Table 14:** Taxonomic list of the small mammals identified in the Hohle Fels Cave assemblage by geological horizon. NISP: number of identified specimens, %: proportion of total material from geological horizon, MNI: minimum number of individuals.

Geological Horizon (GH)		7			7a/7aa			8			9			10			11			12			Grand Total		
Quadrant		30			30			30			30			30			25			25			30 & 25		
Taxonomic category	Common name	NISP	%	MNI	NISP	%	MNI	NISP	%	MNI	NISP	%	MNI	NISP	%	MNI	NISP	%	MNI	NISP	%	MNI	NISP	%	MNI
**Carnivora**																									
*Mustela erminea*	Stoat				1	0.4%	1	6	0.8%	1				1	0.7%	1				1	0.03%	1	9	0.1%	4
**Chiroptera**																									
*Chiroptera* indet.		1	1.5%																	1	0.03%	1	2	0.0%	1
*Myotis* sp.	Mouse-eared bats	1	1.5%	1																1	0.03%	1	2	0.0%	2
**Insectivora**																									
*Talpa* sp.	European moles				1	0.4%	1	1	0.1%	1	4	0.4%	1				1	0.2%	1				7	0.1%	4
Soricidae					1	0.4%		3	0.4%		8	0.7%		1	0.7%		9	2.0%		18	0.5%		40	0.6%	
*Crocidura* sp.											4	0.4%	1										4	0.1%	1
*Crocidura leucodon/russula*	Bicolored/Greater white-toothed shrew				5	1.9%	1	6	0.8%	1	8	0.7%	2										19	0.3%	4
*Crocidura leucodon*	Bicolored shrew				3	1.1%	1																3	0.0%	1
Soricinae								1	0.1%		6	0.5%		6	4.4%		14	3.1%		41	1.2%		68	1.1%	
*Sorex* sp.								5	0.7%	2	1	0.1%	1										6	0.1%	3
*Sorex* cf. *araneus*	Common shrew							1	0.1%	1	4	0.4%	1				1	0.2%	1	48	1.4%	9	54	0.9%	12
*Sorex araneus*	Common shrew	13	19.4%	3	1	0.4%	1	18	2.3%	4	10	0.9%	2	1	0.7%	1	21	4.6%	5	9	0.3%	3	73	1.2%	19
*Neomys* cf. *anomalus*	Miller’s water shrew										3	0.3%	1										3	0.0%	1
*Neomys fodiens*	Eurasian water shrew	2	3.0%	1							4	0.4%	2	2	1.5%	1	4	0.9%	1	10	0.3%	4	22	0.4%	9
**Rodentia**																									
Sciuridae																									
*Spermophilus* sp.																				2	0.1%	1	2	0.0%	1
*Spermophilus superciliosus*	Ground squirrel							1	0.1%	1										3	0.1%	1	4	0.1%	2
Murinae																				1	0.0%	1	1	0.0%	1
Gliridae		1	1.5%	1																			1	0.0%	1
Arvicolinae																									
Arvicola terrestris/antiquus	Water vole				4	1.5%	1	7	0.9%	1	5	0.4%	2	1	0.7%	1	18	3.9%	4	17	0.5%	4	52	0.8%	13
*Dicrostonyx gulielmi*	Collared lemming	2	3.0%	1	10	3.7%	2	26	3.4%	3	14	1.2%	3	2	1.5%	1	5	1.1%	2	53	1.6%	11	112	1.8%	23
*Lemmus lemmus*	Norwegian lemming	4	6.0%	1	36	13.4%	9	76	9.9%	18	76	6.7%	14	6	4.4%	1	19	4.2%	4	307	9.2%	61	524	8.5%	108
*Microtus sp*.		30	44.8%	10	139	51.7%	19	438	57.1%	45	778	68.5%	76	95	69.3%	9	257	56.2%	35	2138	64.1%	301	3875	62.9%	495
*Microtus gregalis*	Narrow-headed vole				15	5.6%	9	45	5.9%	18	59	5.2%	26	3	2.2%	2	8	1.8%	3	133	4.0%	51	263	4.3%	109
*Chionomys nivalis*	Snow vole				5	1.9%	2	7	0.9%	2	5	0.4%	2				8	1.8%	4	9	0.3%	5	34	0.6%	15
*Microtus oeconomus*	Tundra vole	5	7.5%	3	9	3.3%	6	8	1.0%	6	17	1.5%	10	3	2.2%	2	11	2.4%	8	34	1.0%	17	87	1.4%	52
*Microtus subterraneus*	Common pine vole																			2	0.1%	2	2	0.0%	2
*Microtus arvalis/agrestis*	Common/Field vole	8	11.9%	5	39	14.5%	17	118	15.4%	47	129	11.4%	66	16	11.7%	10	81	17.7%	40	505	15.2%	204	896	14.5%	389
Grand Total		67	100%	26	269	100%	70	767	100%	151	1135	100%	144	137	100%	29	457	100%	108	3333	100%	678	6165	100%	1206
NTAXA (richness)				8			12			13			12			9			10			14			19
Reciprocal of Simpsons (1/D)		4.56			5.07			4.63			4.58			4.02			4.27			3.55			4.14		

Arvicolidae is the most numerous group with 9 species represented, including 7 vole species—the water vole (*Arvicola terrestris/antiquus*), narrow-headed vole (*Microtus gregalis*), snow vole (*Chionomys nivalis*), tundra vole (*Microtus oeconomus*), pine vole (*Microtus subterraneous*), and the common (*Microtus arvalis*) and field vole (*Microtus agrestis*). The common and field vole cannot be differentiated by tooth morphology alone, and therefore are presented as one group *Microtus arvalis/agrestis*. The species designation of the *Arvicola terrestris/antiquus* group is left unclear as the maximum length of the specimens from all cultural periods falls within the range of both *A*. *terrestris* and *A*. *antiquus* (S1 Appendix). Ziegler [79] reports a similar pattern in the Geißenklösterle assemblage and distinguishes the two species based on the inferred environment. As the goal of the Hohle Fels analysis is to derive a paleoenvironmental signal from the site, assigning species identifications to these specimens based on assumed climatic conditions would be circular reasoning. Furthermore, the small sample size of *Arvicola* specimens from Hohle Fels (n = 10) leaves open the possibility that the anomalous size is the result of *A*. *terrestris* population variation. This taxonomic dominance by voles is common in Central European Upper Pleistocene assemblages [10,12,80]. The species richness (NTAXA) ranges from 8 to 14 throughout the horizons and exceeds that found by Rhodes et al. [18] at Geißenklösterle. Both cave assemblages are dominated by between 3 and 5 species, with the reciprocal of Simpson’s index (1/D) from the Hohle Fels assemblages ranging from 3.55 to 5.07.

Shrews account for a large proportion of the assemblage from Hohle Fels, even during the Aurignacian when they were rare at Geißenklösterle [18]. This is driven at least in part by the addition of bicolored white-toothed shrews (*Crocidura leucodon* and *C*. *russula*) which occur only in the latest MP and early Aurignacian deposits (GH 9 through 7a/7aa). To our knowledge, this is the only occurrence of white toothed shrews in the Ach Valley, and their preference for dry steppe and open humid woodland environments differs from other Soricinae species identified at the site. The 54 specimens designated *Sorex* cf. *araneus* exhibit a larger than expected size in the lower condylar facet and condylar height (following [43]) and/or molar length and breadth (following [44]). The morphology of these specimens is typical of Soricini, with the lower m1 and m2 exhibiting an entoconid crest and a broad interarticular area of the condyle without lingual emargination, yet their size falls within published measures of *Neomys* sp. from Sesselfelsgrotte [1], Geißenklösterle [18] and Pisede bei Malchin [81]. Storch [12] reported particularly large shrews from Brillenhöhle designated *Sorex* cf. *araneus* and this, combined with the fact that a small number of the Hohle Fels specimens fall within the range of *Sorex* sp. reported by Reumer [43] guided our classification. Although it has been suggested that the large size of the Brillenhöhle *S*. cf. *araneus* specimens was a climatically driven phenotypic response, Prost et al.’s [82] study of soricids from the Pleistocene-Holocene transition in Austria and Belgium found no clear correlation between climate and increased body size in *S*. *araneus* groups.

In addition to voles and shrews we identified the mouse-eared bat (*Myotis* sp.), mole (*Talpa* sp.), and ground squirrel (*Spermophilus superciliosus*) at Hohle Fels. The same species have been identified at other Swabian sites including Geißenklösterle [18,79], Brillenhöhle [12], Hohlenstein [83] and Kogelstein [10]. The specimens attributed to Murinae and Gliridae are both incomplete maxilla fragments identified based on root pattern morphology, and therefore the determinations are considered provisional. However, *Apodemus* sp. was identified from geological horizon 17 at Geißenklösterle [79] and at the MP site of Kogelstein [10]. The single *Talpa* sp. humerus specimen from horizon 8 is missing the proximal portion and part of the diaphysis, restricting our comparison with *T*. *europaea* and *T*. *magna* material to the distal epiphyseal breadth. Based on reports from Villa Seckendorff [84], Hohlenstein [83], and Ochtendung [42] the Hohle Fels specimen falls at the lowest end of the *T*. *(europaea) magna* range and within the average size range of recent southern German populations [1], and so we restricted our identification of all mole material to *Talpa* sp. and placed it into a temperate open forest, cool & moist habitat category in the indicator species paleoenvironmental reconstruction. In the habitat weighting analysis, 10% of the specimen weighting was attributed to cold steppe environment in recognition of the possibility that some of the material may belong to *T*. *(e*.*) magna*.

### Paleoenvironmental signal and its context within the Ach Valley

**Fig 7:**
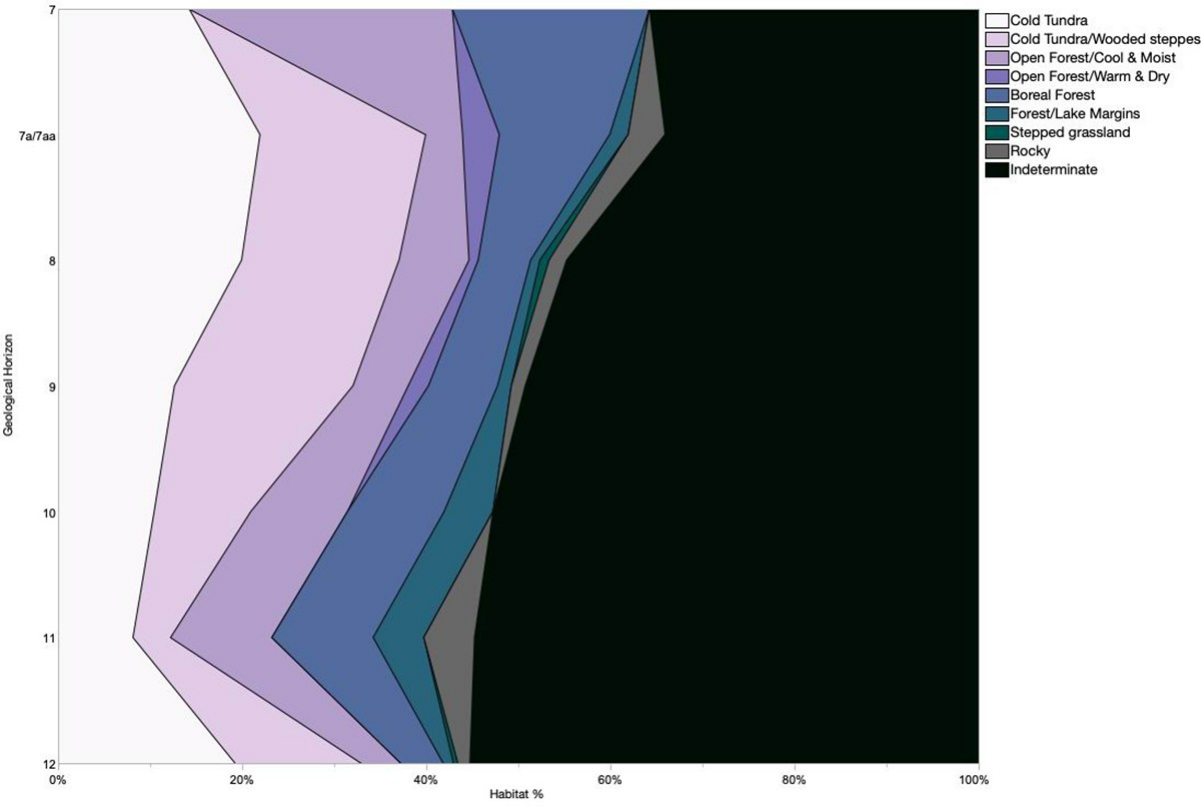
Relative proportion of individuals by habitat types at Hohle Fels Cave by geological horizon.

To assess the paleoenvironmental signal from the Hohle Fels small mammal record we applied both a modified indicator species method [18] and the habitat weighing method [27,28]. The benefit of the indicator species method is that it allows comparison with a number of past small mammal paleoenvironmental studies conducted in Germany and parts of Central Europe [10,12,36,42,83,85,86], including our recent study of the MP and UP small mammal assemblage from Geißenklösterle [18]. We present the results from this method for Hohle Fels in Fig 7. Although a number of similarities in the records from Hohle Fels and Geißenklösterle are immediately apparent, we must be cautious when suggesting one-to-one comparisons as differences in sedimentation rate and time averaging cannot be directly quantified [31]. As such, we discuss these two paleoenvironmental records separately here at the end of this section with the goal of identifying climatic shifts which affected the Ach Valley broadly.

The environmental signal of the earliest layers at Hohle Fels is driven by cold tundra adapted lemmings (35.0%) and various forest adapted species (14.5%), with a small yet important grassland component indicated by the presence of *S*. *superciliosus*. This fits well with the taphonomic data suggesting snowy owls, great grey owls, and European eagle owls accumulated the small mammal material in the early MP, as snowy owls are known to inhabit cold tundra environments and the great grey, European eagle, and snowy owl all hunt within open steppe and forest edge areas [48]. A warm period is indicated by the drop in cold tundra taxa from 33.0% in GH 12 to 12.3% in GH 11, and our results suggest that cold tundra landscapes gradually returned, reaching similar highs only by GH 9 where cold, wooded tundra species account for 32.1% of the assemblage. Our taphonomic analysis suggests that the GH 9 material was likely accumulated by the snowy owl, with inputs from a mammalian predator, likely the arctic fox. This picture of the MP fits well with the micromorphology-based environmental signal from the site, which suggests a warm and wet environment in the lowest most deposits and a decided lack of clearly defined cold stadial/warm interstadial oscillations at Hohle Fels [31]. The ratio of reindeer to other cervids is also at its lowest in the MP of Hohle Fels, suggesting that the environment was warmer and more temperate overall than in the later Aurignacian, with less coniferous forests or stepped environments [87].

At Geißenklösterle, Sirgenstein, and, to a lesser extent, Vogelherd, a hiatus in the occupation of the sites can be clearly seen both stratigraphically [31] and in terms of artifact density. This hiatus is also present at Hohle Fels but is not as clearly defined and constitutes the lower portion of the basal Aurignacian deposits of GH 8 [29,31]. A moderate increase in cold tundra and wooded steppe species can be seen in the Hohle Fels record between GH 9 and GH 8, however, there are no clear indications that a cold snap, a quick and intense cold period, occurred either before, during or directly after the hiatus deposits. Instead, tundra landscapes clearly increase gradually across what is likely a long temporal period. The micromorphological record also suggests that a markedly warm and moist climate defines the lowermost deposits of GH 8, especially when compared to later deposits [31], a signal which may have been obscured in the small mammal record by the occurrence of high proportions of cold adapted taxa in the upper half of GH 8. The presence of the great grey owl, as indicated in the taphonomic record of GH 8, further supports the micromorphological conclusions, as this predator mostly inhabits boreal forest environments and prefers to hunt in open, swampy landscapes [48,88]. The pattern of gradual cooling beginning in GH 9 is also seen in the increasing ratio of reindeer to other cervids in the Hohle Fels faunal record [87] and the increase of willow alongside pine, deciduous birch, and other tundra species in the sites macrobotanical record [89].

The earliest Aurignacian deposits at Hohle Fels includes GH 8 and GH 7, above which the upper Aurignacian (GH 7 – 6a) underlies a phase of mixed Aurignacian and Gravettian deposits [29]. Recently recalibrated ^14^C dates by Bataille and Conard [29] places the earliest Aurignacian to between 41.7 and 39.0 ka calBP with a possible maximum age of 44.0 ka calBP for GH 8. This places the Hohle Fels Aurignacian prior to Heinrich event 4 and raises the possibility that the markedly cold period recognized in the sedimentary and small mammal material directly following the earliest Aurignacian in GH 7a/7aa reflects the onset of H4 in the region. The effect of this cold event extends through GH 7a/7aa and ends with the onset of a warm phase beginning in GH 7. This warming signal is slightly earlier in the stratigraphic chronology than expected, as sedimentary and C^14^ dating place interstadial 7 at GH 6a (directly overlying GH 7). The increasing warm, open forest component seen in the Hohle Fels record in GH 7a/7aa and extending into GH 7 may be indicative of an even earlier start to this pre-H3 warm phase, however this may also be due to time averaging of the deposits and the small sample size (n = 67) of GH 7. Neither the taphonomic signal of GH 7a/7aa, which suggests the presence of the boreal European eagle owl, nor that of GH 7, which best matches actualistic snowy owl assemblages, fits with the overall climatic signals of these horizons. This may be explained by some form of equifinality and/or multi-predator mixed accumulation, although this is not clear from our analysis.

**Fig 8:**
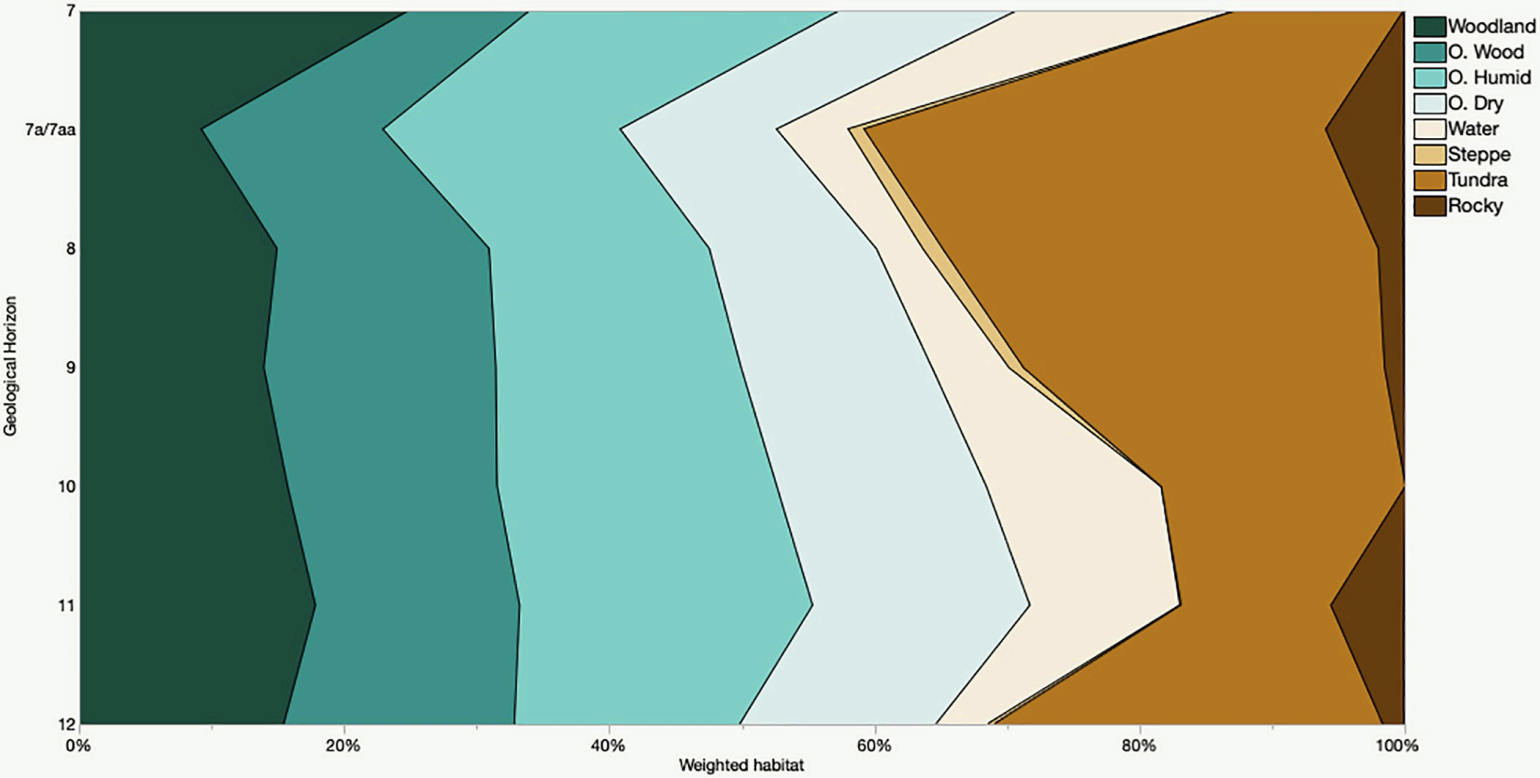
Relative proportion of weighted habitats by geological horizon at Hohle Fels Cave. O. Wood = open wood; O. Humid = open humid; O. Dry = open dry.

We applied the habitat weighting method [27,53] to the small mammal record in an effort to derive a more nuanced picture of the climatic fluctuations throughout the MP to UP transition at Hohle Fels. Comparing the results seen in Fig 8 with those derived from the modified indicator species analysis (Fig 7) we can see the same broad trend of increased cooling throughout all deposits, with a sharp decrease in tundra environments at GH 11 and a subsequent increase in GH 7a/7aa, is present in both records. There is a greater indication of woodland environments during the basal MP deposits in the habitat weighting method results, which fits more closely with the sedimentary signal of a warm and temperate environment during this time [31]. The warm MP event in GH 11 appears to be the result of more lakes and rivers on the landscape as well as open and humid meadows and pastures and increased mature woodland elements. This mosaic landscape remains relatively stable throughout the MP, with only moderate decreases in the open meadow areas coinciding with the first presence of temperate grasslands and the gradually extension of cold, dry tundra landscapes, which increased by close to 20% by the end of the MP (GH 9). The aforementioned marked cold phase of GH 7a/7aa is also clear in the habitat weighing results. That this horizon is defined by the presence of anthropogenic dumped ash features [31] may speak to the increased need for fire for warmth during an extended cold period. A shift back to more temperate conditions is visible in the weighted habitat results of GH 7, with the presence of streams and ponds, humid evergreen meadows, and mature woodlands returning to nearly MP proportions. Overall, the habitat weighting method presents a similar climatic signal to that derived from the modified indicator species analysis, indicating broad agreement between the two paleo-reconstructive methods. This supports our paleoenvironmental reconstruction and suggests that, in this case, the modified indicator species method is not significantly biased by the exclusion of rare and ‘indeterminate’ taxa.

Comparing the small mammal record from Geißenklösterle [18] with this new record from Hohle Fels we note a number of similarities and differences. In the MP rocky and river/lake-edge species are more prominent at Geißenklösterle, however this likely reflects the fact that this site is located high along a steep-sided wall of the Ach Valley, approximately 60 m above the modern valley floor which has likely risen upwards of 40 m since the Paleolithic [31]. At Geißenklösterle the MP is also marked by two warm/moist and cold/dry oscillations recognizable in the MP deposits (between GH 22 and 21, and GH 20 and 19) [18]; events that were also documented in the micromorphological record from this site, which otherwise suggested the Ach Valley was warm and wet at the beginning of the MP [31]. That these climatic oscillations are not seen in the Hohle Fels record may speak to the degree of time averaging inherent in the MP deposits.

At Geißenklösterle the small mammal signal clearly shows a moderate increase in cold tundra and wooded steppe species at the transition from the hiatus layer (GH 17) to the earliest Aurignacian deposits (GH 15/16) similar to the pattern seen at Hohle Fels between GH 9 and GH 8. Here one should note that in contrast to Hohle Fels, where the stratigraphic record over the shift from the late Middle Paleolithic to the Aurignacian is continuous, there is a stratigraphic unconformity in the Geißenklösterle record between the top of GH 17 and the base of the lower Aurignacian layer [31]. Although the stratigraphic observations tell us little about the duration reflected by the unconformity, radiometric dates from Geißenklösterle suggest a duration on the scale of several hundred to a couple thousand years. This being said, the micromorphological work at Geißenklösterle has ruled out the possibility of mixing between Aurignacian and MP deposits [31]. The micromorphological record also suggests that the occupational hiatuses at both sites had markedly warm and moist climates, especially when compared to later deposits [31].

Like the newly calibrated dates from Hohle Fels, ultra-filtration AMS dates from Geißenklösterle place the earliest Aurignacian before the global timing of the Heinrich 4 event [90], suggesting that a similarly markedly cold event seen in both the sedimentary and small mammal records of GH 13 may reflect the onset of this cold period. This cold event extends through GH 12 and, to a lesser extent, GH 11 however the sample sizes available for the small mammal analysis from these upper Aurignacian horizons were quite small and are likely skewing our picture of this period at Geißenklösterle [18]. Overall, these two sites present broadly similar environmental records for the time periods under examination, with differences possibly attributable to variation in sedimentation rate and the effect of time averaging on fine-scale taxonomic variation. As such, we are able to reconstruct a generalized picture of the environment during the MP and UP in the Ach Valley which can then be used to test hypotheses put forth to explain the cultural and biological turnover recognizable in the archaeological record of this region.

## Discussion and conclusions

Based on this analysis of the small mammal material from Hohle Fels we can draw the following conclusions:

1. The small mammal material was most likely accumulated by the snowy owl, European eagle owl, and/or great grey owl, with a limited contribution by foxes. As these predators are mostly generalist hunters, the composition of the assemblages should reflect the diversity of the small mammal community on the landscape at the time.
2. The assemblages are taxonomically rich, comprising 12 genera and 14 distinct species including the first occurrence of the bicolored white-toothed shrew (*Crocidura*) from the Swabian Jura and possible large forms of *Arvicola* and *Talpa*. Rarefaction analysis suggests that only two or three rare species are missing from each GH examined.
3. There is a clear trend of increased cooling and the spread of tundra and steppe environments from the MP through the early Aurignacian, with a warm event in GH 11 and a cold event in GH 7a/7aa suggested by both the modified indicator species and habitat weighting methods.
4. The habitat weighting method indicates that mature woodlands, open meadows, and lakes and rivers were prominent components of the MP landscape.
5. There is no clear signal for drastic climatic change before or during the cultural hiatus. Therefore, climate should not be invoked as the driving force behind the depopulation of the region by Neanderthal groups.

These results echo other paleoenvironmental signals derived from the macrobotanical [89], micromorphological [31], and large fauna [87] records from Hohle Fels and the surrounding sites. By applying two paleoenvironmental-reconstructive methods to the Hohle Fels material, this study follows other recent multi-analytic small mammal studies [80,91] in attempting to minimize the influence of both rare and abundant taxa–each of which affect small mammal reconstructive methods to different degrees [91]. With the incorporation of a taphonomically rigorous small mammal climatic signal, we can confidently surmise that the MP of the Ach Valley was a mosaic of dry tundra and open coniferous and old-growth forests, with rivers and ponds close to the cave. A period of climatic amelioration occurred around the middle of the MP record, allowing the spread of forests, pastures, and grasslands, likely associated with an increase in precipitation and water sources in the valley. Overall, the environmental signal at Hohle Fels suggests a more homogenously temperate climate than that indicated from similar material records at other sites [18].

Importantly, this study further supports the conclusion that the MP to UP transition, including the nearly-culturally sterile ‘hiatus’ found at Geißenklösterle and Hohle Fels caves, saw a gradual increase in cold and dry arctic environments [18]. This likely correlates with the cooling trend spanning D-O cycles 12–9 seen elsewhere in Western Europe [25]. That the reaction of the Swabian small mammal community to the D-O cycles and related Heinrich events is less pronounced than we would expect suggests that the Ach Valley may have experienced a regionally bound ecological response to these climatic episodes, similar to what has been suggested for the Lower Danube region [92]. As none of the environmental proxies studied in detail (which include the small mammal, botanical, and sedimentary records) have revealed indications of a ‘cold snap’ or increased climatic instability around the MP to UP transition, the hypothesis that climatic variability lead to the abandonment of the region by Neanderthal groups as put forth in variants of the *Kulturpumpe* and *Population Vacuum* models cannot be substantiated. While the *Kulturpumpe* model also posits that inter-taxa competition and/or internal socio-economic dynamics may have triggered the cultural innovations of the Swabian Aurignacian [93], the presence of ‘hiatus’ deposits between the two cultural periods in the stratigraphy of many sites in the region, as well as the lack of transitional lithic industries, suggests that inter-taxa competition and/or acculturation were not active factors within the Swabian Jura.

However, the current study does suggest that the earliest Swabian UP groups arrived in the region during a particularly cold and dry period, when the landscape was dominated by cold tundra and wooded steppe landscapes. That these groups remained and flourished in such a challenging environment suggests that UP cultural and symbolic innovations may have arisen in part as a response to the gradual climatic cooling during the early Aurignacian [93,31]. In this context, one should recall that figurative art, musical instruments and personal ornaments are well documented from the basal Aurignacian horizons [6, 14], which suggests that they were part of the behavioral repertoire of the Aurignacian inhabitants of Swabia when they arrived in the region, or that they developed nearly immediately after they entered this region that appears to have been largely void of indigenous Neanderthal populations. One argument for the local evolution of these cultural features is the complete absence of mammoth ivory figurines, bone flutes, three dimensionally formed ivory ornaments and other specific elements of the Swabian Aurignacian lithic and organic technologies in other European regions at this early date.

Concerning the late Middle Paleolithic of the Swabian Jura, a wide range of observations indicate that population densities were typically much lower than during the Aurignacian and that small highly mobile and perhaps demographically isolated groups of Neanderthals occupied the region [74]. In Central Europe as a whole, Neanderthal groups appear to have lived in small and genetically isolated yet self-sufficient and highly mobile populations [94]. Within this framework, Neanderthals may have adapted to climatic shifts by retreating out of and later recolonizing areas affected by stadial and interstadial oscillations [25,95]. Furthermore, it is highly likely that some groups experienced local extinction events in the face of climatic pressures or inter-species competition [3]. Comparison of the density of anthropogenically derived materials, including lithic artifacts, burnt bone, charcoal and modified fauna, through time does suggest that Neanderthals occupied the Swabian sites with lower intensity or for shorter durations than later Aurignacian groups [18,31,74]. The more ephemeral nature of Neanderthal site use in this region may reflect real differences in population size as well as mobility and subsistence strategies, which may have proven detrimental both genetically [95] and in terms of innovative potential [96] in the face of the climatic instability of OIS 3 and the arrival of larger and more socially connected modern human groups. Interbreeding and niche competition with modern humans was clearly a factor in the extinction of Neanderthals across Europe [21], however these inter-species interactions did not contribute to the loss of Neanderthal populations within the Swabian Jura. Furthermore, as this study has shown, dramatic climatic instability did not drive Neanderthal groups from the Ach Valley [18,31]. The gradual decline in the environmental hospitality of the region may help to explain why Neanderthal groups left, as one of possibly many retreats [25] within a cycle of repeated abandonment and recolonization of Northern latitude regions. Isotopic studies have shown that individual Neanderthals travelled upwards of 20 km during their lifetime [97], and evidence for long distance movement of raw materials within the Swabian Jura suggest greater ranges of resource exploitation [98,99]. The stadial periods recognizable in the marine isotopic records would have evolved on a decadal scale [4], allowing for generational recognition of the changing landscapes and resources due to increased aridity and lowering temperatures across southern Germany. Recognition of the gradually declining climatic conditions may have prompted the late MP Swabian Neanderthal groups to abandon the region before facing local extinction. While speculative in nature, new and ongoing studies from within the Ach Valley and nearby areas will hopefully allow for further testing of this hypothesis explaining the decline of Neanderthal populations and the dramatic appearance of modern humans during the early Aurignacian of the Upper Danube region.

## Supporting information

S1 Appendix Table 1 Inferior m1 length of Arvicola from Hohle Fels cave and other key sites; Table 2: Cranial elements by geological horizon and bucket ID number; Table 3: Post-cranial elements and incisors by geological horizon and bucket ID number. (XLSX)
